# Comprehensive Transcriptome Analysis of mRNA Expression Patterns Associated With Enhanced Biological Functions in Periodontal Ligament Stem Cells Subjected to Short-Term Hypoxia Pretreatment

**DOI:** 10.3389/fgene.2022.797055

**Published:** 2022-02-08

**Authors:** Zhi-Bang Li, Hui-Qi Yang, Kun Li, Yuan Yin, Su-Su Feng, Shao-Hua Ge, Yang Yu

**Affiliations:** ^1^ Shandong Key Laboratory of Oral Tissue Regeneration & Shandong Engineering Laboratory for Dental Materials and Oral Tissue Regeneration, Department of Periodontology, School and Hospital of Stomatology, Cheeloo College of Medicine, Shandong University, Jinan, China; ^2^ State Key Laboratory of Military Stomatology, National Clinical Research Center for Oral Diseases, School of Stomatology, Fourth Military Medical University, Xi’an, China; ^3^ Department of Periodontology, Jinan Stomatological Hospital, Jinan, China

**Keywords:** periodontal ligament stem cells, normoxia, hypoxia, RNA-seq, genes, biological functions

## Abstract

Short-term hypoxia pretreatment significantly enhances periodontal ligament stem cell (PDLSC)-based periodontal tissue regeneration by improving various cellular biological functions, but the underlying mechanisms remain unclear. In this study, based on RNA sequencing (RNA-seq), we comprehensively analyzed the possible regulatory mechanisms of the short-term hypoxic effects on the biological functions of healthy and inflammatory PDLSCs. A total of 134 and 164 differentially expressed genes (DEGs) were identified under healthy and inflammatory conditions, respectively. Functional enrichment analyses indicated that DEGs under both conditions share certain biological processes and pathways, including metabolic processes, developmental processes, reproductive processes, localization, immune system processes and the HIF-1 signaling pathway. The DEGs identified under inflammatory conditions were more significantly enriched in cell cycle-related processes and immune-related pathways, while DEGs identified under healthy condition were more significantly enriched in the TGF-β signaling pathway. A protein-protein interaction network analysis of the 59 DEGs in both conditions was performed, and 15 hub genes were identified. These hub genes were mainly involved in glycolysis, the cellular response to hypoxia, cell differentiation, and immune system processes. In addition, we found that hypoxia induced significant differential expression of genes associated with proliferation, differentiation, migration, apoptosis and immunoregulation under both healthy and inflammatory conditions. This study provides comprehensive insights into the effects of short-term hypoxia on the biological functions of PDLSCs and suggests a potentially feasible strategy for improving the clinical effectiveness of cell-based periodontal tissue engineering.

## 1 Introduction

Periodontitis is a complex infectious disease that can lead to loss of connective tissue and bone support, which results in the formation of periodontal pockets around teeth (Pihlstrom et al., 2005). Unfortunately, severe periodontitis can cause tooth loosening, pathological migration, and eventually tooth loss in most instances, and these effects undoubtedly result in a decrease in quality of life (Slots, 2017; Gugliandolo et al., 2021). The basic principle of periodontal therapy involves controlling biofilm formation and other contributing factors, deescalating inflammation, and regenerating damaged periodontal tissue. In this regard, the clinical outcomes of reduced tissue inflammation and pocket depths can be achieved by conventional nonsurgical therapy, effective self-care and, in some cases, the administration of local or systemic antibiotics (Pihlstrom et al., 2005; Slots, 2017). For patients with severe periodontitis, the use of various surgical procedures, including guided tissue/bone regeneration, produces periodontal regeneration to varying degrees (Kassebaum et al., 2014). However, true structural and functional regeneration of the alveolar bone, cementum, and periodontal ligament cannot be achieved using these traditional methods. Although the local or systemic administration of biomaterials and growth factors regenerates damaged periodontal support, clinical trials have revealed that this approach produces limited effects, and these findings reveal that true periodontal regeneration remains an ongoing challenge for dentists (Xu Q. et al., 2019).

In this context, to overcome the limits of the low regenerative capacities of biomaterials or growth factors, the combined/individual application of stem cells provides a promising strategy for regenerative medicine (Marconi et al., 2020; Gugliandolo et al., 2021). Specifically, mesenchymal stem cell (MSC)-based therapy has been demonstrated to be an effective approach for tissue repair and/or regeneration due to the remarkable multilineage differentiation property, anti-inflammatory potential, immunomodulatory function and autocrine secretion of these cells (Daley and Scadden, 2008; Parekkadan and Milwid, 2010; Najar et al., 2016). Indeed, many MSC populations derived from bone marrow, adipose tissue, umbilical cord, and dental tissues (including dental pulp, apical papilla, periodontal ligament, dental follicle, gingiva, and alveolar bone) have exhibited tremendous therapeutic potential for periodontal regeneration (Liu D. et al., 2012; Pezzi et al., 2017). Based on several small-scale pilot/feasibility studies, due to their tissue specificity, periodontal ligament stem cells (PDLSCs) hold great promise for regenerating the periodontal complex, which consists of appropriate levels of alveolar bone, PDL tissue and new cementum production along with the root surface (Chen et al., 2016; Park et al., 2016; Xu Q. et al., 2019).

To obtain sufficient cell aggregation, isolated stem cells are subjected to a long-term *ex vivo* expansion procedure. However, the current *in vitro* culture systems cannot mimic the desired *in vivo* environment, which resulting in the weakening and even loss of cell stemness, particularly with regard to proliferation, migration, differentiation and immunoregulation (Yu et al., 2016a; Jayaraman et al., 2016). Hence, many strategies have been developed to maintain or enhance the stem cell biological functions during *ex vivo* expansion and to improve their regeneration potential (Teramatsu et al., 2014; Marconi et al., 2020; Gugliandolo et al., 2021). Some promising results for improving the regenerative potential of MSCs have been achieved by genetic modification or pretreatment with cytokines (Gugliandolo et al., 2021). However, the accompanying drawbacks such as biosafety risks and expensive pretreatment costs have hindered the clinical use of these approaches.

As demonstrated by various studies, the hypoxia pretreatment of cells appears to be a simple, safe, feasible and effective method because the oxygen concentration in the *in vivo* niche containing MSCs varies from 1% to more than 7% (Boyette et al., 2014; Lee et al., 2015). In this regard, our previous studies demonstrated that a short-term (less than 48 h) low-oxygen atmosphere (e.g., 2% oxygen concentration) exerts a positive effect on the migration of both bone morrow MSCs (BMMSCs) and PDLSCs, which particularly improves the *in vitro* behavior and *in vivo* periodontal regenerative potential of PDLSCs (Yu et al., 2016a; Yu et al., 2016b).

In addition to maintaining or improving the regenerative potential of PDLSCs under hypoxic conditions, exploring the underlying mechanisms is needed to establish an optimal *ex vivo* expansion system. Indeed, some studies have demonstrated that the exposure of PDLSCs to hypoxia could promote their osteogenic potential, mineralization and paracrine release through the mitogen-activated protein kinase kinase/extracellular signal-regulated kinase (MEK/ERK) and p38 MAPK signaling pathways (Wu et al., 2013). Similarly, Xu Q et al. found that hypoxia could mediate the expression of Runt-related transcription factor 2 (RUNX2) in PDLSCs via hypoxia-inducible factor-1α (HIF-1α)-induced vascular endothelial growth factor (VEGF) action and may play a positive role in the early stage of osteogenesis driven by PDLSCs (Xu Q. et al., 2019). Additionally, the clone formation and proliferation capacities of PDLSCs are enhanced under hypoxic conditions via activation of the p38/MAPK and ERK/MAPK signaling pathways (He et al., 2016). Some studies have indicated that circCDK8 represses the osteogenic differentiation of PDLSCs by triggering autophagy activation in hypoxic microenvironments (Zheng et al., 2021). Nevertheless, the understanding of the impact of hypoxia on periodontal tissues and the biochemical mechanism of action remains immature and rudimentary, and the underlying mechanism warrants further investigation.

Currently, with the rapid development of high-throughput RNA sequencing (RNA-Seq) technology, the whole transcriptome changes in eukaryotes under different conditions have been identified, and this information provides progressively greater knowledge of both the quantitative and qualitative aspects of transcript biology and reveal the potential transcriptional mechanisms of various diseases (Ozsolak and Milos, 2011; Demircioglu et al., 2019). In the present study, we performed RNA-seq-based transcriptome analyses to identify the core dynamic signature of differentially expressed genes (DEGs) whose expression is affected by the short-term hypoxia pretreatment of cells derived from healthy and inflammatory PDL tissues. The results reveal the overarching alteration of gene expression in PDLSCs under hypoxia and provide experimental evidence showing that PDLSC pretreatment leads to a robust increase in cellular products for clinical use in periodontal regenerative medicine.

## 2 Materials and Methods

### 2.1 Human Subjects and Ethics

RNA-seq of extracted tooth samples obtained from 6 human bodies, including three subjects with healthy periodontal tissue (the healthy condition) and three subjects with periodontitis (the inflammatory condition), was performed. All subjects were generally healthy, without systemic diseases and had not received antibiotics or other prescribed medications in the 3 months prior to tooth extraction. Periodontitis was diagnosed following the consensus report of Workgroup 2 of the 2017 World Workshop (Berglundh et al., 2018). All subjects were recruited from Shandong University Dental Hospital and signed written informed consent forms to participate. This study was approved by the Medical Ethics Committee of the Stomatology School at Shandong University.

### 2.2 Cell Isolation, Culture and Identification

Human PDLSCs (hPDLSCs) were collected and cultured as described previously (Yu et al., 2016a; Marconi et al., 2020). We scraped tissue from the middle 1/3 root of each extracted tooth, collected these tissues in a centrifuge tube, and added 1 ml of type I collagenase (3 mg/ml) and 1 ml of neutral protease (4 mg/ml) to the centrifuge tube. The centrifuge tube was placed in an incubator for 1 h (37°C with 5% CO2), and the digestion was terminated by the addition of α-MEM containing 10% fetal bovine serum (FBS) to the centrifuge tube. The centrifuged precipitant contained the hPDLSCs, which were inoculated in 6-well plates and cultured in 5% CO2 at 37°C. The medium was refreshed every 3 days. Once the cell monolayer reached 80% confluence, hPDLSCs were trypsinized and passaged. To characterize the mesenchymal features of isolated hPDLSCs, the expression of cell surface antigens was determined by flow cytometric analysis and their differentiation potential was evaluated via osteogenic and adipogenic differentiation assays as described previously (Yu et al., 2016a; Marconi et al., 2020).

hPDLSCs at passage 3 (P3) were randomly assigned to the normoxia group (cultured in 20% O2 and 5% CO2 at 37°C) and the hypoxia group (cultured in 2% O2 and 5% CO2 at 37°C). Hypoxic conditions were induced in a hypoxia incubator (Thermo Fisher Scientific, Waltham, MA, United States). Three biological replicates of all the samples were established, and 12 h later, the hPDLSCs were lysed and used for performed RNA-seq analysis.

### 2.3 RNA-Seq Analysis

Total RNA was isolated as previously described (Yu et al., 2016a). The mRNA was enriched by oligo dT selection, generated into short fragments in fragmentation buffer, reverse transcribed into first-strand cDNA with random N6 primers, and synthesized into double-stranded cDNA using first-strand cDNA as the template. After end repair, the double-stranded cDNA was amplified by polymerase chain reaction (PCR) to construct a cDNA library. RNA-seq was performed using a BGISEQ-500 platform, and the read length was 150 bp.

The sequencing data were filtered using SOAPnuke software (version 1.5.0). The clean filtered reads were aligned to the human reference genome (GRCh38) using HISAT software. Fragments per kilobase per million map reads (FPKM) values were calculated to evaluate the expression level. Differentially expressed genes (DEGs) were identified using DESeq2. The significant DEGs were defined based on a Q value (adjusted *p* value) < 0.05 and absolute log2-fold change (FC) ≥ 1. The volcano map and expression heatmap were generated using online analysis tools (https://www.omicstudio.cn/tool).

### 2.4 Annotation and Functional Analysis of the DEGs

We performed functional analyses of the DEGs under healthy and inflammatory conditions. We conducted a Gene Ontology (GO) functional enrichment analysis and KEGG pathway enrichment analysis using the Dr. Tom program (https://biosys.bgi.com) provided by BGI Tech (BGI-Shenzhen, China), and Q values <0.05 were considered to indicate significant DEG enrichment. A protein-protein interaction (PPI) network analysis was conducted using the STRING database (https://www.string-db.org/) and Cytoscape software (version 3.8.2). Significant clusters and hub genes in the PPI network were identified using the Molecular Complex Detection (MCODE) and CytoHubba plug-ins in Cytoscape. The functions of the genes in the PPI network and the clusters were further analyzed using the Metascape database (Zhou et al., 2019), and the results are displayed as a heatmap of enriched terms. A KEGG pathway enrichment analysis was performed, and the outputs were categorized using the ClueGO plug-in (version 2.5.8) in Cytoscape. The pathways were categorized into functional groups based on a kappa score of 0.4 and visualized in a KEGG pathway network diagram with the associated genes. In addition, we identified DEGs associated with proliferation, differentiation, migration, apoptosis, and immunoregulation by performing a PPI network analysis, and three hub genes were identified for each of the five aforementioned functions.

To analyze the similarities and differences in the functions and pathways of the DEGs under healthy and inflammatory conditions, enrichment analyses were conducted using the Metascape database. For the DEGs common to both healthy and inflammatory conditions, we performed GO and KEGG enrichment analyses and then performed pathway and process enrichment analyses using Metascape. After performing a PPI network analysis, we grouped the pathway and process terms into clusters and identified significant modules and hub genes. The KEGG pathway enrichment analysis was also performed using the ClueGO plug-in in Cytoscape, and the results were visualized in a KEGG pathway network diagram with the associated genes.

### 2.5 Gene Set Enrichment Analysis

A GSEA was performed using the Dr. Tom program (https://biosys.bgi.com) to investigate the significant biological functions and pathways. In this analysis, all the genes (not solely DEGs) were considered and gene-gene correlations were identified (Subramanian et al., 2005).

### 2.6 Quantitative Real-Time PCR

Five DEGs were selected to verify the reliability of the RNA-seq data by quantitative real-time PCR (qRT-PCR). The relative mRNA expression levels were calculated using the 2^–ΔΔCt^ method after normalization to GAPDH expression. The primers were designed using Primer-BLAST, which is available on the NCBI website. Each gene was assessed in three biological replications. The qRT-PCR data were statistically analyzed by Student’s t-test, and are expressed as the means ± SE. The threshold for significance was set to *p* value <0.05.

## 3 Results

### 3.1 Summary of the Transcriptome Profiles

The hPDLSCs derived from all the donors exhibited a long spindle-like morphology and were positive for the MSC markers CD90, CD146 and STRO-1 but negative for the hematopoietic markers CD34 and CD45 (Supplementary Figure S1). All the cells derived from the different individuals exhibited the capacity to differentiate into osteogenic and adipogenic lineages *in vitro* (Supplementary Figure S1). Our RNA-seq analysis was performed with 12 samples; and from each sample an average of 6.83 G worth of data were obtained, and 24,961 genes were detected. The average ratio of the sample comparison genome was 92.19%, and the average alignment of the gene set was 84.08%. All Pearson correlation coefficients between two samples were higher than 0.85 (Supplementary Figure S2), which indicated high correlations. The 12 samples shared a fairly consistent distribution of gene expression levels (Supplementary Figure S3) and a consistent expression density distribution (Supplementary Figure S4).

### 3.2 Identification and Functional Analysis of the DEGs Under Healthy Condition

#### 3.2.1 Identification of DEGs

**FIGURE 1:**
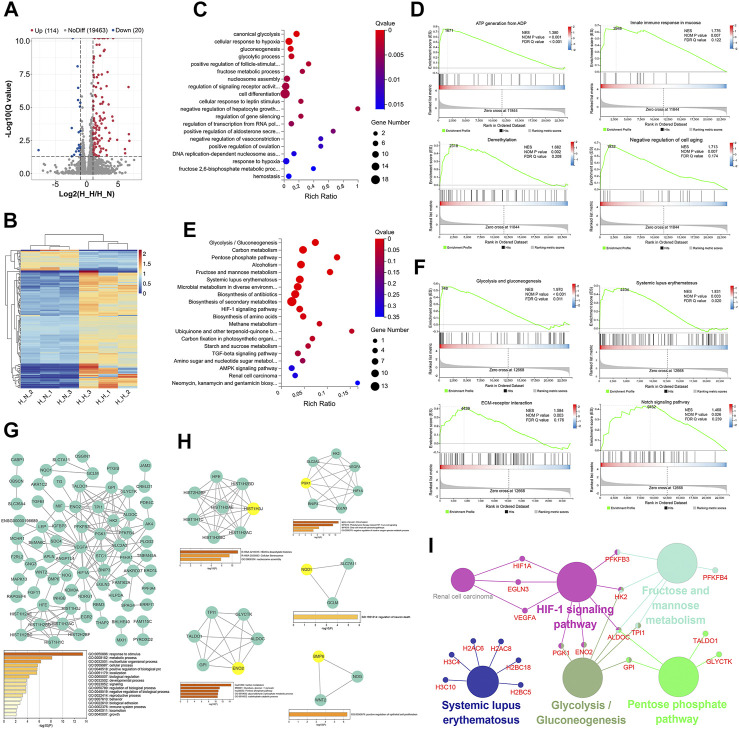
Identification and functional analysis of differentially expressed genes (DEGs) in normoxia-treated healthy hPDLSCs (H_N) compared with hypoxia-treated healthy hPDLSCs (H_H). **(A)** Volcano map of DEGs analyzed by DESeq2 (|log2 FC| ≥ 1; Q value≤0.05); the red dots represent upregulated DEGs, the green dots represent downregulated DEGs, and the gray dots represent genes that did not show significant differential expression. **(B)** Expression heatmap of each sample with the expression standardized by log (expression+1); each row represents a gene, each column represents a sample, and the colors in the panels represent the relative expression levels, with blue indicating low expression and red indicating high expression. **(C)** GO enrichment analyses of the DEGs. **(D)** Gene set enrichment analysis (GSEA) of biological processes, including ATP generation from ADP, innate immune response in mucosa, demethylation, and negative regulation of cell aging. **(E)** KEGG enrichment analyses of the DEGs. **(F)** GSEA of KEGG, including glycolysis/gluconeogenesis, systemic lupus erythematosus, ECM-receptor interaction and the Notch signaling pathway. **(G)** PPI network of the DEGs and the three most highly correlated hub genes. **(H)** Significant modules in the PPI network and the most highly correlated hub gene in each module; the hub genes are shown in yellow. **(I)** Network of KEGG pathway enrichment results determined by ClueGO; the large circles are pathways, their size depends on the *p* value, and the small circles are the genes found in the pathway.

The comparison of healthy hPDLSCs under normoxia (H_N) with healthy hPDLSCs under hypoxia (H_H) identified a total of 134 DEGs, including 114 upregulated and 20 downregulated DEGs (Figure 1A, Supplementary Table S2). The expression of the DEGs in each sample is shown in an expression heatmap (Figure 1B). The gene expression levels were generally comparable between the two groups, and showed good repeatability (Supplementary Figure S5). The average FPKM values of the DEGs in each sample in the hypoxia group were higher than those in the normoxia group.

#### 3.2.2 GO and KEGG Enrichment Analyses

The top 20 significantly enriched GO terms are shown in Figure 1C and Supplementary Table S2. The DEGs were enriched in various GO terms including canonical glycolysis, cellular response to hypoxia, gluconeogenesis, positive regulation of follicle-stimulating hormone secretion, and cell differentiation. A GSEA of the DEGs identified 62 processes that were significantly enriched, and these included ATP generation from ADP, innate immune response in mucosa, demethylation, and negative regulation of cell aging (Figure 1D, Supplementary Table S3).

Fourteen KEGG pathways were significantly enriched, and these included glycolysis/gluconeogenesis, the pentose phosphate pathway (PPP), systemic lupus erythematosus, the HIF-1 signaling pathway, and amino acid biosynthesis (Figure 1E, Supplementary Table S4). Similar results were also found from the GSEA: 10 pathways, including glycolysis and gluconeogenesis, systemic lupus erythematosus, ECM-receptor interaction and the Notch signaling pathway, were significantly enriched (Figure 1F, Supplementary Table S5).

#### 3.2.3 PPI Network and Clusters

The PPI network of the 134 identified DEGs is shown in Figure 1G, and the most enriched GO biological processes identified using Metascape are shown below the network in the figure. The 134 DEGs were significantly enriched in metabolic process, developmental process, multicellular organismal process, reproductive process, biological adhesion, locomotion, and immune system process. These findings indicate that these DEGs may be related to the proliferation, differentiation, migration, apoptosis and immunoregulation of hPDLSCs. The three hub genes with the highest correlations were HFE, HIST1H2AC, and HIST1H2BD.

Five clusters (Figure 1H) associated with cellular senescence, the HIF-1 signaling pathway, glucose metabolism, positive regulation of epithelial cell proliferation, and regulation of neuron death were identified in this network. The top hub gene for each of the five modules was HIST1H3J, PGK1, ENO2, BMP6, and NQO1.

#### 3.2.4 KEGG Pathway Network

The ClueGO KEGG pathway enrichment analysis revealed that five functional groups were significantly enriched (Figure 1I). The DEGs under healthy condition were mainly involved in glycolysis and gluconeogenesis, the PPP, fructose and mannose metabolism, the HIF-1 signaling pathway, and systemic lupus erythematosus.

#### 3.2.5 Five Key Regeneration-Related Functions

**FIGURE 2:**
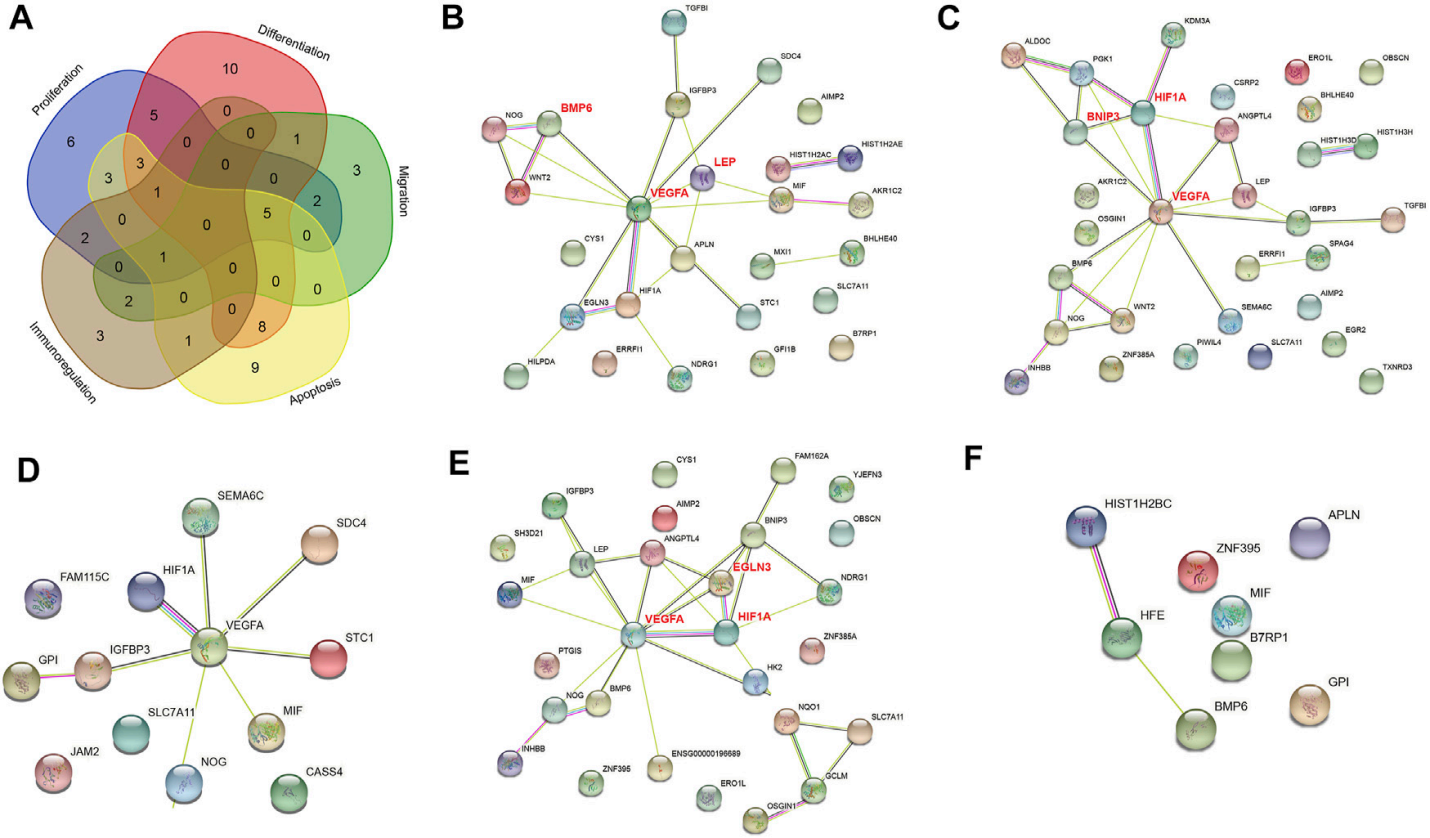
PPI networks of differentially expressed genes (DEGs) in normoxia-treated healthy hPDLSCs (H_N) compared with hypoxia-treated healthy hPDLSCs (H_H). **(A)** Venn diagram of DEGs associated with proliferation, differentiation, migration, apoptosis, and immunoregulation. PPI networks of DEGs associated with proliferation **(B)**, differentiation **(C)**, migration **(D)**, apoptosis **(E)**, and immunoregulation **(F)**. The genes in bold red are the three hub genes with the highest correlation as identified by CytoHubba.

To identify the mechanism of the effect of hypoxia on cell tissue regeneration potential under healthy condition, we performed PPI network analyses with DEGs associated with proliferation, differentiation, migration, apoptosis, and immunoregulation (Figure 2). All five networks interacted at significant levels (PPI enrichment *p* value <0.05). The DEGs enriched in the proliferation, differentiation, migration and apoptosis networks were significantly enriched in the HIF-1 signaling pathway (FDR <0.05). The differentiation and apoptosis network-related DEGs were significantly enriched in the TGF-beta signaling pathway (FDR <0.05). Six hub genes, namely, VEGFA, HIF1A, BMP6, BNIP3, EGLN3, and LEP, were identified in the proliferation, differentiation and apoptosis networks. No edges or hub genes were identified in the migration or immunity network.

### 3.3 Identification and Functional Analysis of DEGs Under Inflammatory Condition

#### 3.3.1 Identification of DEGs

**FIGURE 3:**
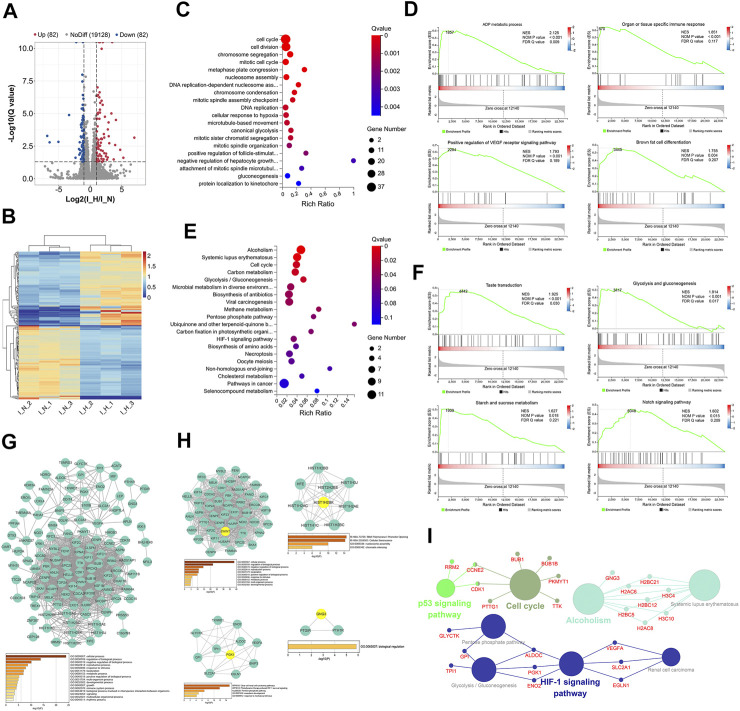
Identification and functional analysis of differentially expressed genes (DEGs) in normoxia-treated inflammatory hPDLSCs (I_N) compared with hypoxia-treated inflammatory hPDLSCs (I_H). **(A)** Volcano map of DEGs analyzed by DESeq2 (|log2 FC| ≥ 1; Q value≤0.05); the red dots represent upregulated DEGs, the green dots represent downregulated DEGs, and the gray dots represent genes showing nonsignificant differences in expression. **(B)** Expression heatmap of each sample with the expression standardized by log (expression+1); each row represents a gene, each column represents a sample, and the colors in the panels represent the relative expression levels, with blue indicating low expression and red indicating high expression. **(C)** Gene Ontology (GO) enrichment analyses of the DEGs. **(D)** Gene set enrichment analysis (GSEA) of biological processes, including ADP metabolic processes, organ or tissue specific immune responses, positive regulation of the vascular endothelial growth factor receptor (VEGFR) signaling pathway, and brown fat cell differentiation. **(E)** Kyoto Encyclopedia of Genes and Genomes (KEGG) enrichment analyses of the DEGs. **(F)** GSEA of KEGG pathways identified, including taste transduction, glycolysis/gluconeogenesis, starch and sucrose metabolism, and the Notch signaling pathway. **(G)** PPI network of the DEGs and the three most highly correlated hub genes. **(H)** Significant modules of the PPI network and the hub gene showing the highest correlation with each module; the hub genes are shown in yellow. **(I)** Network obtained from the ClueGO KEGG pathway enrichment analysis; the large circles are pathways, their size is dependent on the *p* value, and the small circles are genes indicated in the pathway.

The comparison of the hypoxia group (I_H) and the normoxia group (I_N) identified 164 DEGs, including 82 upregulated and 82 downregulated DEGs (Figure 3A, Supplementary Table S6). The DEG expression level in each sample is shown in an expression heatmap (Figure 3B). The gene expression levels were generally comparable between the two groups, and the data showed good repeatability (Supplementary Figure S6). The average FPKM values of the DEGs in each sample of the hypoxia group were higher than those in the normoxia group.

#### 3.3.2 GO and KEGG Enrichment Analyses

The top 20 significantly enriched GO terms are shown in Figure 3C and Supplementary Table S7. These DEGs were significantly enriched in 55 GO terms, including the cell cycle, canonical glycolysis, cell proliferation, and the innate immune response in mucosa. Similar results were found from the GSEA, which revealed that 55 processes, including the ADP metabolic process, the organ- or tissue-specific immune response, positive regulation of the VEGF receptor signaling pathway, and brown fat cell differentiation, were significantly enriched with DEGs (Figure 3D, Supplementary Table S8).

Thirteen KEGG pathways were significantly enriched with DEGs, and these included alcoholism, systemic lupus erythematosus, the cell cycle, glycolysis/gluconeogenesis, and the HIF-1 signaling pathway (Figure 3E, Supplementary Table S9). Similar results were found from the GSEA, which revealed that 10 pathways, including taste transduction, glycolysis and gluconeogenesis, starch and sucrose metabolism, and the Notch signaling pathway, were significantly enriched (Figure 3F, Supplementary Table S10).

#### 3.3.3 PPI Network and Clusters

The PPI network of the 164 DEGs is shown in Figure 3G. The most enriched GO biological processes identified using Metascape are shown below the network in the figure, and these included developmental process, reproductive process, multiorganism process, localization, and immune system process. These findings indicated that these DEGs may be related to the proliferation, differentiation, migration, apoptosis and immunoregulation of inflammatory hPDLSCs. The three hub genes with the highest correlations in the PPI were KIF11, KIF23, and BUBIB.

Four clusters (Figure 3H) associated with cell division, cellular senescence, mesoderm development, and biological regulation were identified in this network. The top hub gene of each of the five modules was ZWINT, HIST1H2BK, PGK1, and GNG3.

#### 3.3.4 KEGG Pathway Network

The ClueGO KEGG pathway enrichment analysis revealed that four functional groups were significantly enriched (Figure 3I). The DEGs identified under inflammatory condition were mainly involved in the cell cycle, alcoholism, the HIF-1 signaling pathway, and the p53 signaling pathway.

#### 3.3.5 Five Key Regeneration-Related Functions

**FIGURE 4:**
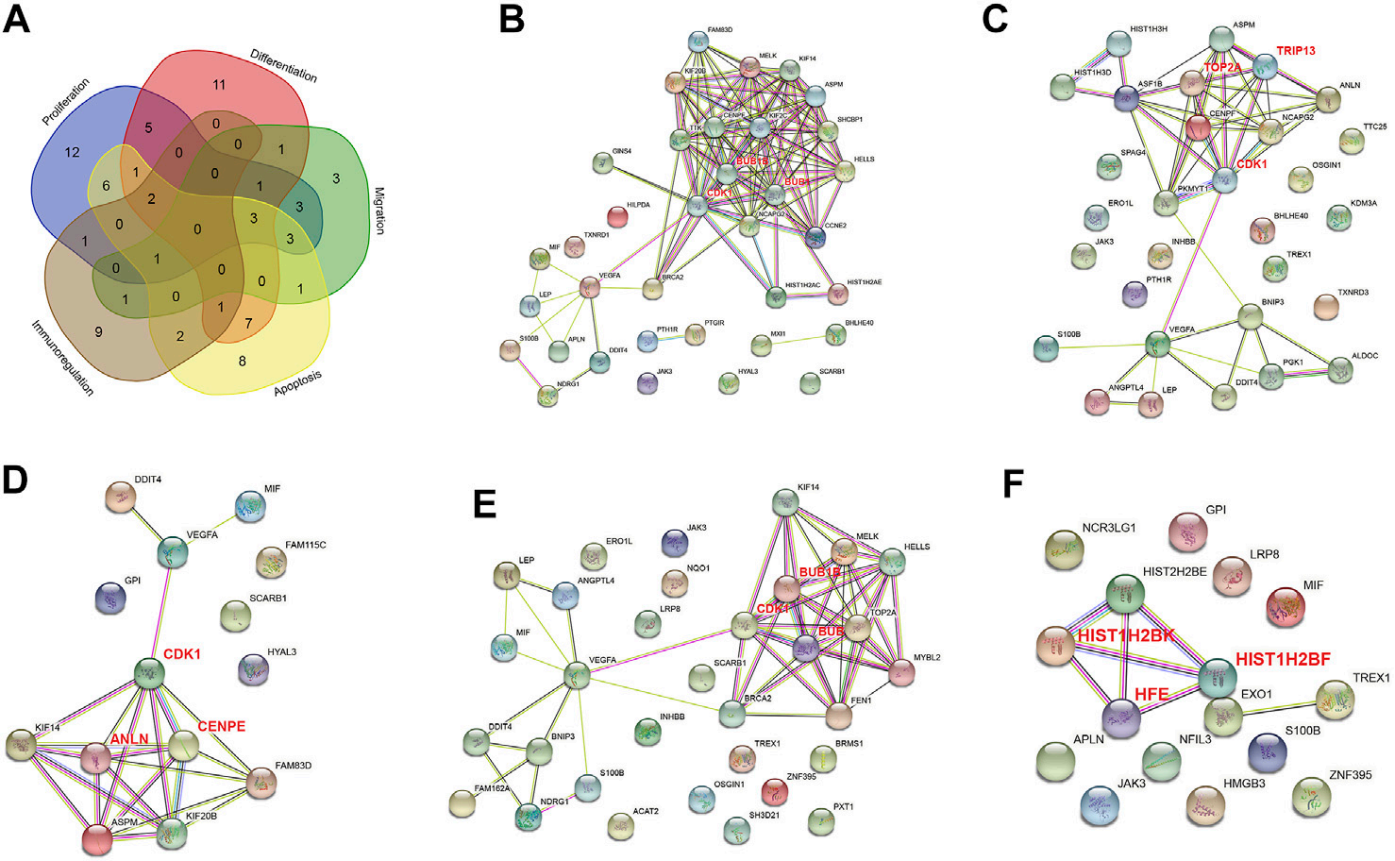
PPI networks of differentially expressed genes (DEGs) in normoxia-treated inflammatory hPDLSCs (I_N) compared with hypoxia-treated inflammatory hPDLSCs (I_H). **(A)** Venn diagram of DEGs associated with proliferation, differentiation, migration, apoptosis, and immunoregulation. PPI networks of DEGs associated with proliferation **(B)**, differentiation **(C)**, migration **(D)**, apoptosis **(E)**, and immunoregulation **(F)**. The genes in bold red are the three most highly correlated hub genes identified by CytoHubba.

To identify the mechanism through which hypoxia affects the tissue regeneration potential under inflammatory condition, we performed a PPI network analysis of DEGs associated with proliferation, differentiation, migration, apoptosis, and immunoregulation (Figure 4). All five networks interacted at significant levels (PPI enrichment *p* value <0.05). Three hub genes were identified for each cluster, and these were CDK1, BUB1, BUB1B, TOP2A, TRIP13, ANLN, CENPE, HFE, HIST1H2BF, and HIST1H2BK.

### 3.4 Similarities and Differences in Processes and Pathways Under Healthy and Inflammatory Conditions

**FIGURE 5:**
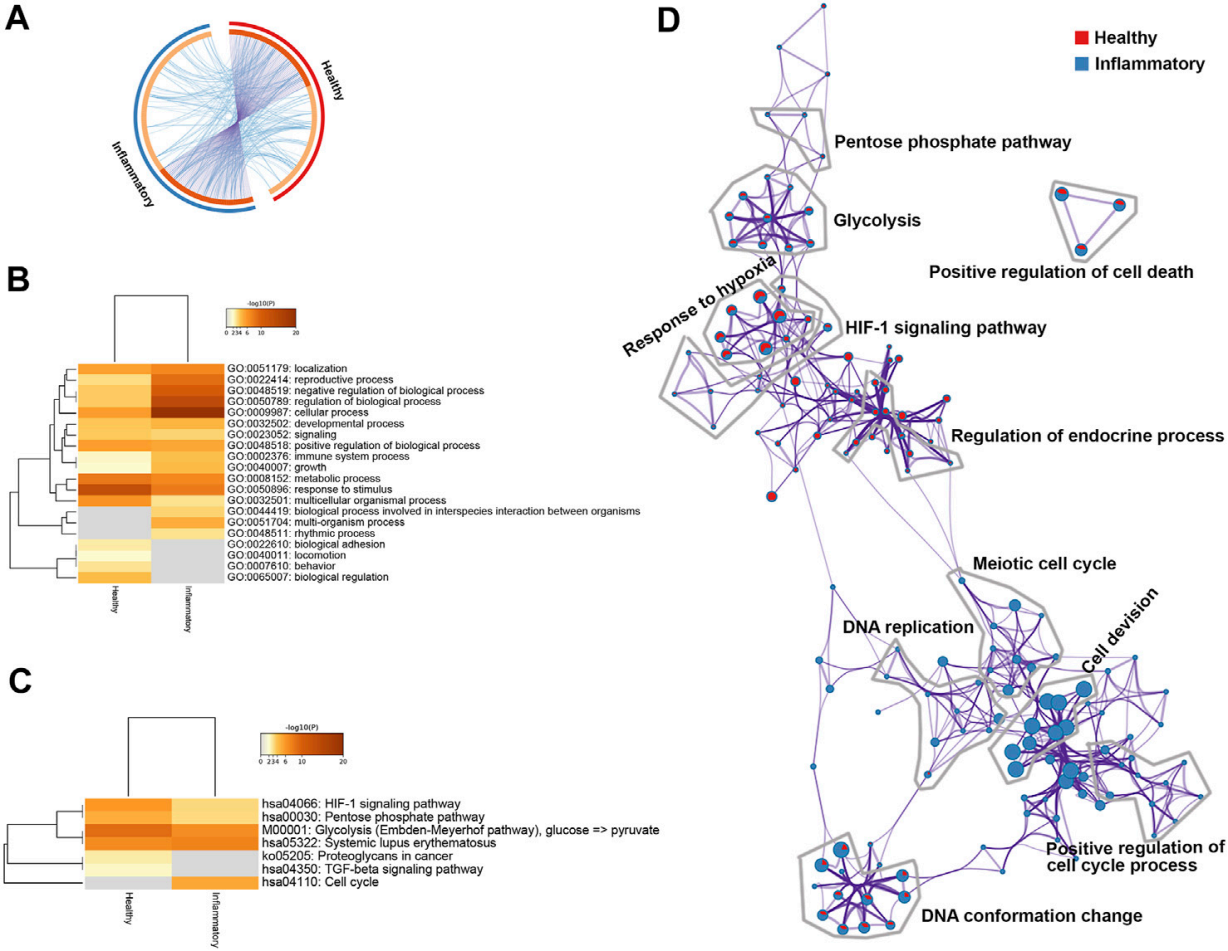
Differentially expressed genes (DEGs) in healthy or inflammatory hPDLSCs. **(A)** Circos plot showing the overlap between the DEGs under the two conditions. **(B)** Most enriched Gene Ontology (GO) biological processes determined using Metascape. The processes are colored according to the *p* value. **(C)** Heatmap of enriched Kyoto Encyclopedia of Genes and Genomes (KEGG) pathways determined using Metascape. The processes are colored according to the *p* value. **(D)** Network of enriched terms in pie charts. The red color represents enriched terms under healthy condition and blue color represents enriched terms under inflammatory condition.

A broad overlap was found among the functions and pathways involving the DEGs under the healthy condition and those under the inflammatory condition (Figure 5A). The DEGs under both conditions were common in certain biological processes, including the response to stimulus, metabolic processes, developmental processes, reproductive processes, localization, and immune system processes (Figure 5B). In contrast, biological adhesion and locomotion were significantly enriched only under the healthy condition, and multiorganism processes and rhythmic processes were significantly enriched only under the inflammatory condition. The enriched pathways common to both conditions were glycolysis, systemic lupus erythematosus, the PPP and the HIF-1 signaling pathway (Figure 5C). In contrast, TGF-β was significantly enriched only under the healthy condition, whereas the cell cycle was significantly enriched only under the inflammatory condition. The 100 biological processes and pathways that were most enriched are presented, and notably, the p53 signaling pathway was significantly enriched under the inflammatory condition (Supplementary Figure S7).

The results from pathway and process enrichment analyses based on both GO biological processes and KEGG pathways are displayed in a network (Figure 5D). The network clearly showed that the following terms were enriched under both healthy and inflammatory conditions: response to hypoxia, the HIF-1 signaling pathway, glycolysis, the PPP and regulation of endocrine process. Cell cycle-related terms were significantly enriched under the inflammatory condition, and these terms included cell division, meiotic cell cycle, DNA replication, and positive regulation of cell cycle processes.

### 3.5 Common Differentially Expressed Genes Under Healthy and Inflammatory Conditions

**FIGURE 6:**
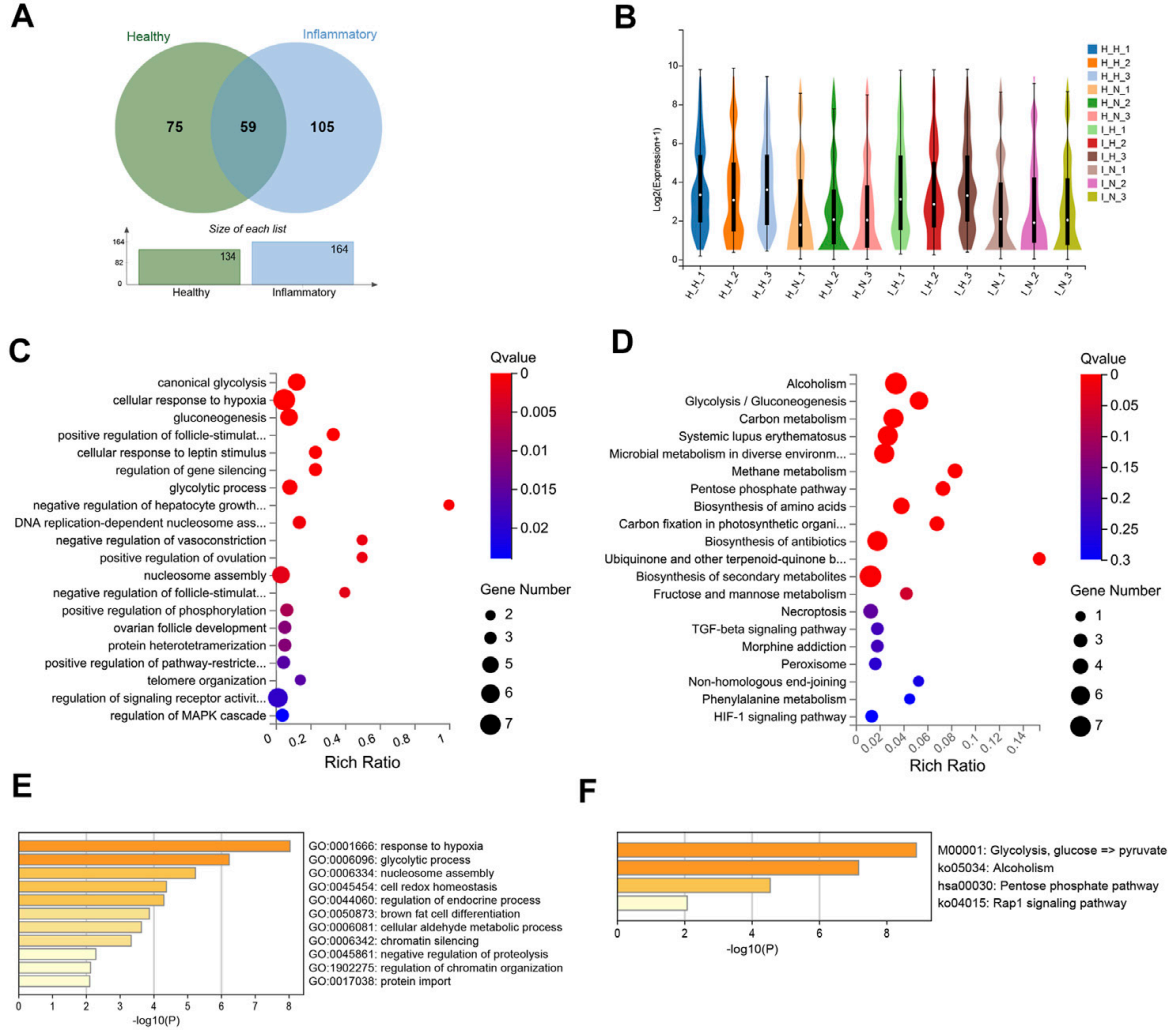
Differentially expressed genes (DEGs) in both healthy and inflammatory hPDLSCs. **(A)** Venn diagram of the common DEGs. **(B)** Violin plot of gene expression for each sample. **(C)** Gene Ontology (GO) enrichment analyses of the DEGs. **(D)** Kyoto Encyclopedia of Genes and Genomes (KEGG) enrichment analyses of the DEGs. **(E)** Bar graph of the enriched KEGG pathways determined using Metascape. The pathways are colored according to the *p* value. **(F)** Bar graph of enriched GO biological processes identified using Metascape.

A total of 59 DEGs were identified under both conditions (Figure 6A, Supplementary Table S11), and these DEGs included 53 upregulated and 6 downregulated DEGs (Figure 6B).

These 59 DEGs were significantly enriched in biological processes related to canonical glycolysis, the cellular response to hypoxia, cell differentiation, negative regulation of the apoptotic process, positive regulation of the p38 MAPK cascade, and the interleukin-7-mediated signaling pathway (Figure 6C). Twelve KEGG pathways were significantly enriched, and these included alcoholism, glycolysis/gluconeogenesis, systemic lupus erythematosus, and biosynthesis of amino acids (Figure 6D). The results from the enrichment analysis of biological processes (Figure 6E) and pathways (Figure 6F) performed using the Metascape database were similar to those described above.

The PPI network of the 59 DEGs constructed using STRING and Cytoscape (Supplementary Figure S8A). Significant processes and pathways, including glycolysis, the response to hypoxia, alcoholism, and the Rap1 signaling pathway, were identified (Supplementary Figure S8A). Two modules associated with cellular senescence and glycolysis were identified in this network (Supplementary Figure S8B). Among these 59 genes, 20 with the greatest differential expression were considered potential core genes. Fifteen genes, including TPI1, MIF, VEGFA, ANGPTL4, and ERO1A, were found to be linked to each other in additional PPI network analyses and were identified as core genes (Supplementary Figure S8C).

The KEGG pathway network of these 59 DEGs was also constructed (Supplementary Figure S9). Three functional pathways, including glycolysis/glycogenesis, the PPP, and alcoholism, were found to be significantly enriched.

### 3.6 Validation of the RNA-Seq Data

**FIGURE 7:**
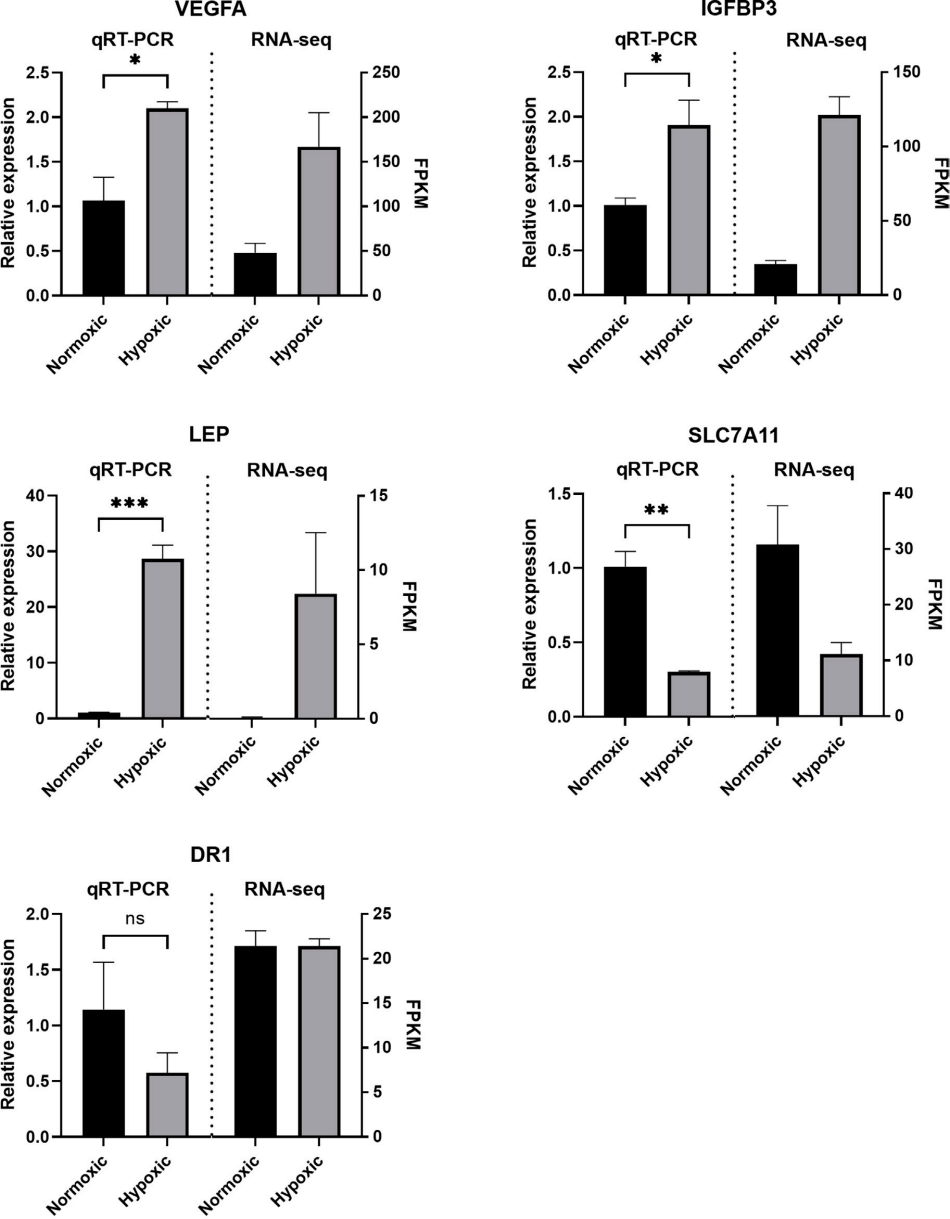
Validation of the RNA sequencing (RNA-seq) data. The quantitative real-time PCR (qRT-PCR) results showed the same trend as the RNA-seq data. The relative mRNA expression levels were calculated using the 2^–ΔΔCt^ method after normalization to GAPDH expression. The data are expressed as means ± SE. *, *p* < 0.05; **, *p* < 0.005; ***, *p* < 0.0005.

To assess the reliability of the RNA-seq data obtained in this study, we randomly selected five genes and performed real-time quantitative PCR to verify their relative expression levels in periodontal stem cells. Three genes (VEGFA, IGFBP3, and LEP) were selected from the set of upregulated DEGs, one gene (SLC7A11) was selected from the set of downregulated DEGs, and one gene (DR1) was selected from the set of nonsignificantly changed genes. The relative expression levels obtained from the qRT-PCR analysis followed the same trends as those indicated by the RNA-seq data, which implied that the sequencing data were valid and reliable (Figure 7).

## 4 Discussion

### 4.1 Summary of the Findings

Our study showed that hypoxia exerted a significant effect on the gene transcription of hPDLSCs. We identified the pathways and key genes involved in the effect of hypoxia on the function of hPDLSCs, revealed the regulatory mechanisms of oxidative stress, and analyzed the commonalities and differences in the mechanisms of hypoxia between healthy and inflammatory hPDLSCs. This study helps reveal the mechanism of the effect of hypoxia on hPDLSC functions and may facilitate interventions into the biological processes of stem cells in culture for improved stem cell therapy.

### 4.2 Common Mechanisms Under Healthy and Inflammatory Conditions

In the present study, the DEGs under healthy and inflammatory conditions were enriched in some common biological processes and pathways. This commonality suggests that hPDLSCs respond in a similar manner to hypoxia under these two conditions. The common biological processes significantly enriched with DEGs included developmental processes, reproductive processes, multicellular organismal processes, localization, and positive regulation of cell death. This finding suggests that hypoxia may affect the entire life process of stem cells, including their proliferation, differentiation, migration and apoptosis. This supposition is consistent with the results of our study and other studies. In our previous study, we found that short-term hypoxia promoted the migration and proliferation of hPDLSCs *in vitro* and enhanced osteogenesis *in vivo* periodontal defect model after hPDLSC transplantation (Yu et al., 2016b). Other studies have also shown that short-term exposure to hypoxia can significantly affect hPDLSC functions, including proliferation (Wu et al., 2013), osteogenic differentiation (Wu et al., 2013; Zheng et al., 2021), mineralization (Wu et al., 2013; Yu et al., 2016a), osteogenesis (Xu X.-Y. et al., 2019), and apoptosis (Zheng et al., 2021).

Certain enriched pathways are common to cells under both healthy and inflammatory states. These pathways may play key roles in hypoxia by regulating biological processes of hPDLSC. One of the significantly enriched pathways was the HIF1 signaling pathway, and the expression of the hub genes (VEGFA, PGK1, and ENO2) associated with this pathway was significantly increased under hypoxia. Significantly enriched clusters associated with the HIF1 signaling pathway were also present under both healthy and inflammatory conditions. These results suggest that the HIF1 signaling pathway plays a key role in the hPDLSC response to hypoxia. This outcome is consistent with the findings of previous studies. The HIF-1 signaling pathway plays an integral role in the response of stem cells to hypoxia. The contribution of HIF1A to the transcriptional response driven by acute hypoxia is particularly important (Lee et al., 2019). In hPDLSCs, hypoxia-induced HIF1A was previously found to regulate the expression of VEGF, RUNX2 and TGF-β1 (Xu X.-Y. et al., 2019; Liu et al., 2019), which is consistent with our results. In addition, HIF-1α (HIF1A) is known to be associated with the adaptation of tissue metabolism to hypoxia, the upregulation of glycolytic gene expression, anaerobic ATP synthesis, and the upregulation of adenosine receptor expressions (Lee et al., 2019). VEGF is a downstream target gene in the HIF-1 signaling pathway and is important for improving the oxygen supply to hypoxic regions (Lee et al., 2019). In our study, VEGF expression was high under both healthy and inflammatory conditions and was significantly increased under hypoxic conditions. VEGF is a core gene in the network of genes associated with proliferation, differentiation, and migration under healthy condition. It has been confirmed that hypoxia enhances the protein expression of VEGF *in vitro* and *in situ* (Xu X.-Y. et al., 2019). Under hypoxia, VEGF regulates the expression of RUNX2 and enhances osteocalcin in hPDLSCs at the early stage of osteogenesis (Xu X.-Y. et al., 2019). All of these results suggest that hypoxia triggers the activation of the HIF signaling pathway and affects stem cell function by elevating the expression of downstream VEGF.

Under both healthy and inflammatory conditions used in this study, DEGs were also significantly enriched in biological process pathways related to metabolism, mainly the glycolysis and gluconeogenesis pathway and PPP. This finding suggests that short-term hypoxic stimulation enhances the metabolic processes in hPDLSCs and that enhanced energy consumption is mainly provided by enhanced gluconeogenesis and glycolysis. Stem cell metabolism is currently considered as a key determinant of cellular processes (Rigaud et al., 2020). Under different environmental stimuli (including hypoxia), stem cell metabolic processes switch to adapt and drive various biological processes. It has been shown that hypoxia shifts hPDLSC metabolism from oxidative phosphorylation to glycolysis and enhances cell proliferation, migration and osteogenic differentiation (Mao et al., 2020). This hypoxia-induced metabolic reprogramming is essential to meet the cellular energy requirements during acute hypoxic stress. The PPP also plays an important role in the cellular processes and stemness maintenance of MSCs under hypoxia. The PPP is a metabolic pathway parallel to glycolysis that provides pentose phosphate and nicotinamide-adenine dinucleotide phosphate (NADPH) for nucleic acid synthesis and plays a pivotal role in cell survival and growth (Jin and Zhou, 2019). In addition, the metabolic shift toward glycolysis and PPP activation is accompanied by increased cell stemness (Liu et al., 2017; Shyh-Chang and Ng, 2017). Therefore, we hypothesize that hypoxia induces hPDLSC metabolism switching from oxidative phosphorylation to glycolysis and activation of the PPP and thus provides more energy and metabolic resources for accelerated biological processes. The related pathways are the glycolysis pathway, PPP, and HIF-1 signaling pathway.

### 4.3 Differences in the Mechanisms Between Healthy and Inflammatory Conditions

One notable difference identified in this study was that inflammatory hPDLSCs were more significantly enriched in cell cycle related items, including cell division, cell proliferation and cellular senescence. Interestingly, the expression of cell cycle-related genes, such as PTTG1, ANLN, and BUB1, was significantly decreased. The P53 signaling pathway, which is associated with cell senescence, was also more significantly enriched and the transcription of the P53-associated genes RRM2, CDK1, and CCNE2 was significantly reduced. These outcomes suggest that inflammatory insult suppresses the cell cycle including apoptosis in addition to hypoxic stimuli. The clinically observed effect of hypoxia on promoting the proliferation of periodontal stem cells under inflammatory condition may be related to a prolonged cell life cycle (Yu et al., 2016a).

Another difference between the two tested conditions was that DEGs under inflammatory condition were significantly enriched in immune-related pathways. This difference may be related to the impact of inflammation on the immune functions regulated by hPDLSCs. Previous studies have shown that inflamed hPDLSCs exert a diminished inhibitory effect on T cell proliferation compared with that exerted by healthy cells (Liu Y. et al., 2012; Zhu and Liang, 2015). Another possible reason for this difference may involve the effect of hypoxia on the immunoregulatory function of inflamed periodontal stem cells. Recent studies suggest that hypoxia pretreatment may affect the secretome of periodontal membrane stem cells (Giacoppo et al., 2017). hPDLSCs, which have low immunogenicity and potent immunomodulatory capacity (Shang et al., 2021), inhibit the proliferation of allogeneic T cells and suppress B cell proliferation, differentiation, and migration through cell-to-cell contact (Queiroz et al., 2021). The effect of hypoxia on the immunomodulatory function of hPDLSCs sheds light on their potential application in periodontitis immunotherapy.

Another interesting difference was that the TGF-β signaling pathway was significantly enriched, and the expression of its core gene, TGFBI, was significantly increased under healthy condition, but no significant changes were found under inflammatory condition. TGF-β1 plays important roles in cell growth, differentiation and transformation. As observed in a previous study, exogenous TGF-β1 decreases the proliferation of periodontal stem cells (Lim et al., 2020). TGF-β induces hPDLSC senescence by increasing reactive oxygen species (ROS) production (Fan et al., 2019) and inhibits periodontal membrane stem cell osteogenesis in synergy with HIF-1α (Liu et al., 2019). Appropriate inhibition of TGF-β1 expression may provide a therapeutic basis for the treatment of periodontitis (Fan et al., 2019). Therefore, the decrease in TGF-β1 expression may partially explain the elevated proliferation and differentiation capacities of inflamed hPDLSCs. Hypoxia-induced suppression of TGF expression is very important for the treatment of periodontitis.

## 5 Conclusion

In this study, we constructed differentially expressed RNA profiles by RNA-seq and demonstrated that gene expression was significantly changed under hypoxic conditions in both healthy and inflammatory hPDLSCs. Oxidative stress regulates stem cell function by affecting the transcription of genes that play important roles in the adaptation of stem cells to hypoxic environments. Hypoxia affectes the entire life cycle of both healthy and inflammatory hPDLSCs, including their proliferation, differentiation, migration and apoptosis. Hypoxia may also affect the immunomodulatory functions of hPDLSCs. The HIF1 signaling pathway and metabolic switching play a key role in the hPDLSC response to hypoxia, and hypoxia may inhibit the TGF-β pathway and activate the P53 signaling pathway in inflammatory stem cells. In summary, the findings obtained in this study provide strong evidence for elucidating the mechanisms underlying the effects of short-term pretreatment on hPDLSCs, and thus further offer potential intervention targets for improving the regenerative efficiency of transplanted cells.

## Data Availability

The datasets presented in this study can be found in online repositories. The names of the repository/repositories and accession number(s) can be found below: GSA-Human: HRA001565; at https://ngdc.cncb.ac.cn/gsa-human.
