# Supplementary material for: Comprehensive Transcriptome Analysis of mRNA Expression Patterns Associated With Enhanced Biological Functions in Periodontal Ligament Stem Cells Subjected to Short-Term Hypoxia Pretreatment
Source: Front Genet. 2022 Feb 8;13:797055. doi: 10.3389/fgene.2022.797055 (PMC8861432; doi:10.3389/fgene.2022.797055)
Supplement: Supplementary file 1 [file DataSheet1.docx]

Supplementary Material

#
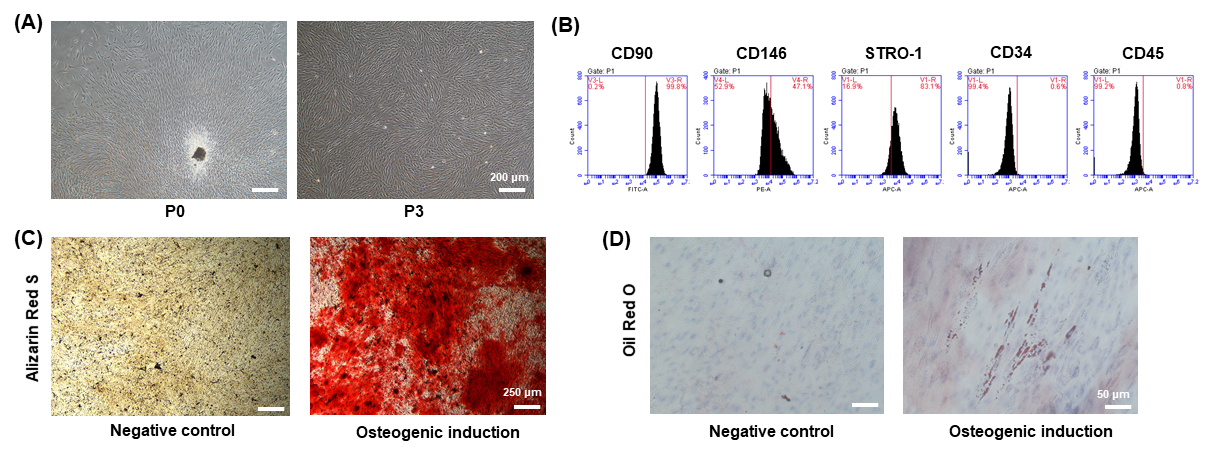
Supplementary Figures

**Figure S1.** Identification of human hPDLSCs. (A) Morphological images of hPDLSCs at P0 and P3. (B) Cell surface markers identified by flow cytometric analysis. (C, D) Representative images of mineralized cell nodules (histochemically stained with Alizarin Red) and of lipid droplets (stained with Oil Red O).


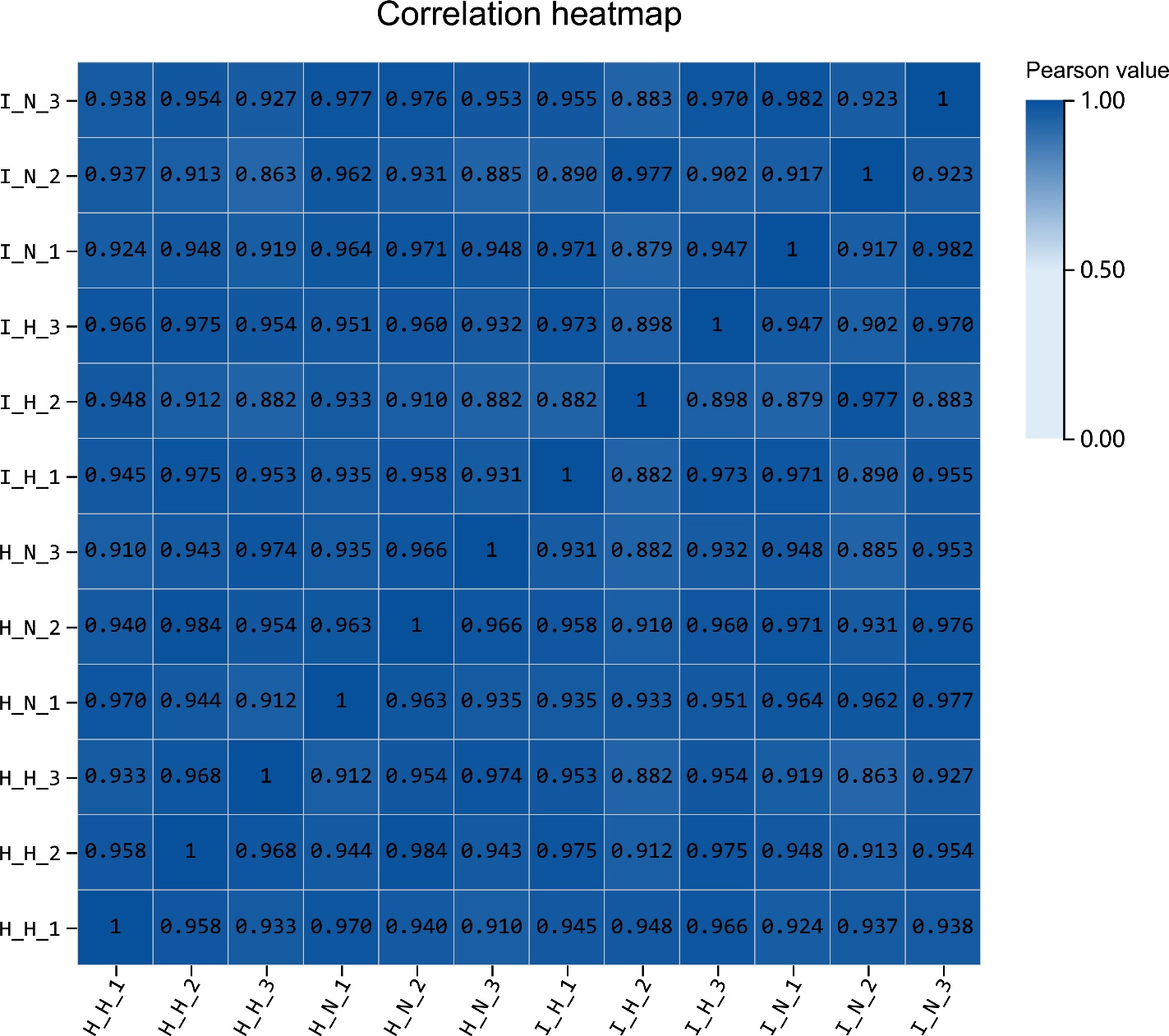


**Figure S2.** Sample correlation heat map. The X- and Y-axes represent each sample. The number in the box is the Pearson correlation coefficient of all genes expressed between each pair of samples. Color represents the correlation coefficient (a darker color represents a higher correlation), and a correlation coefficient higher than 0.85 indicates a high correlation.


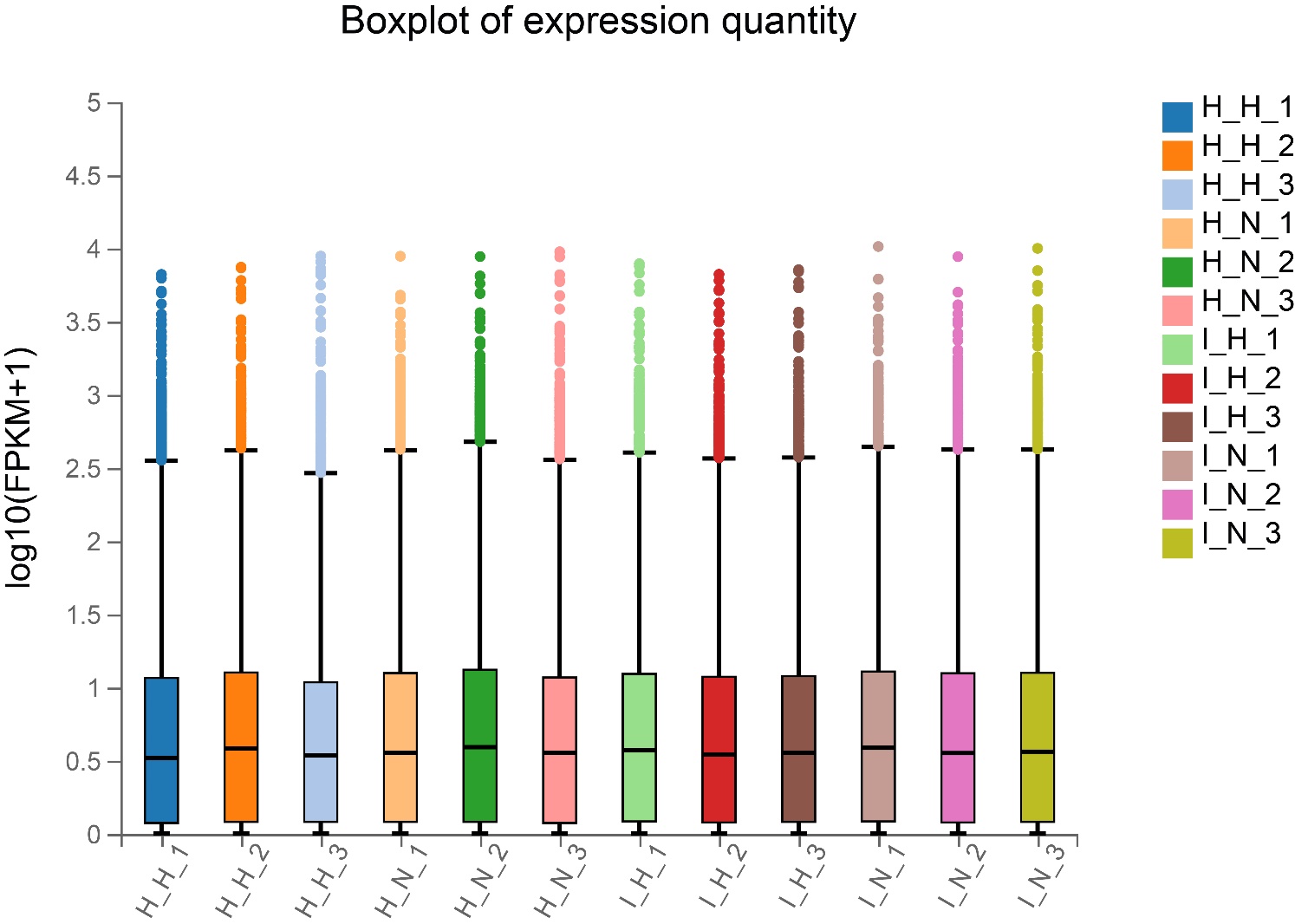


**Figure S3.** Boxplot of expression quantity. The X-axis shows the sample name, and the Y-axis shows log10 (FPKM + 1). The boxplot of each area corresponds to five statistics (the maximum, upper quartile, median, lower quartile, and minimum). FPKM, fragments per kilobase per million map reads.


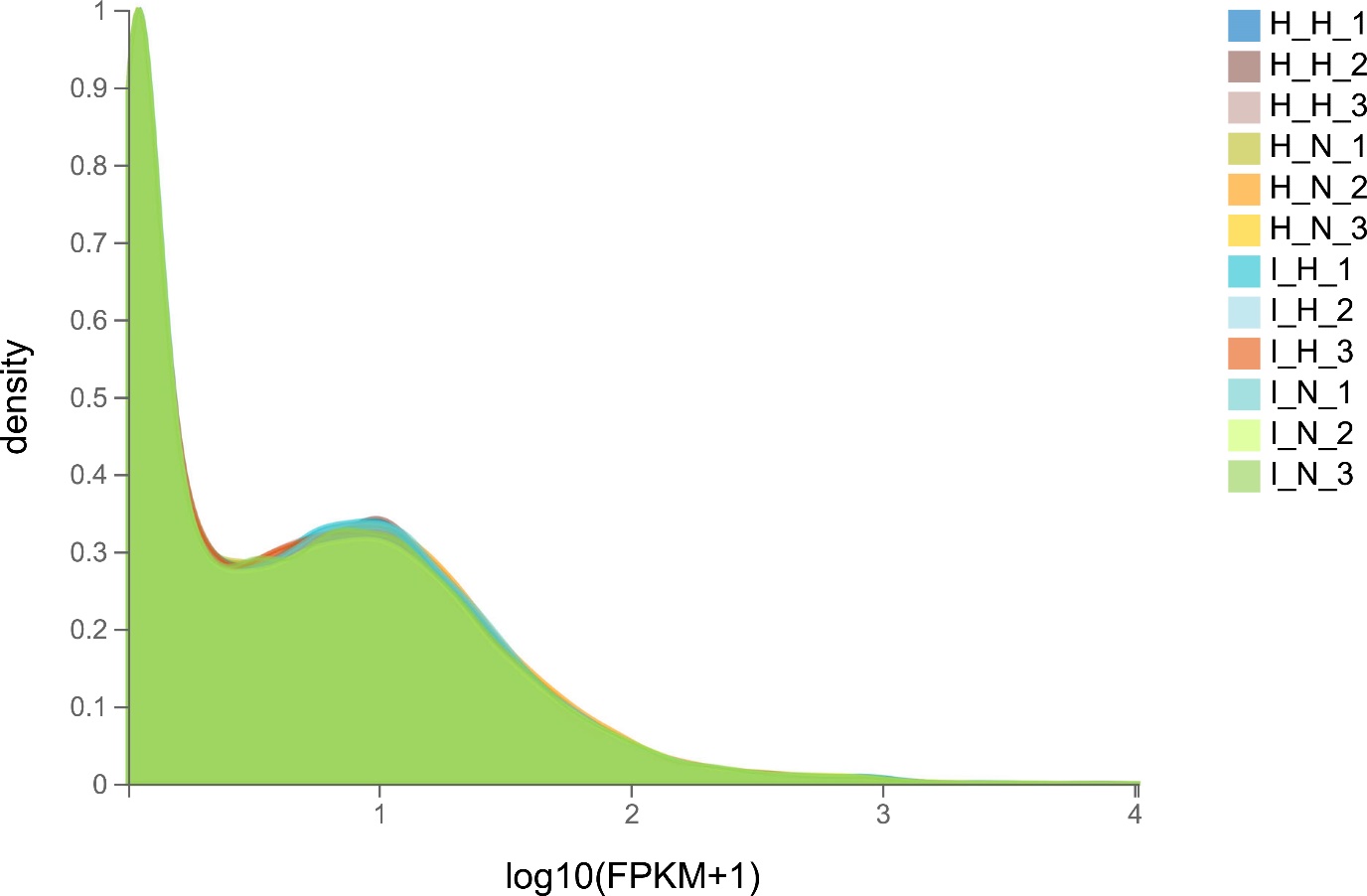


**Figure S4.** Density map of expression quantification distribution. The X-axis shows log10 (FPKM + 1). The Y-axis shows the gene density; that is, the ratio of the number of genes to the total number of genes expressed is this expression quantity. FPKM, fragments per kilobase per million map reads.


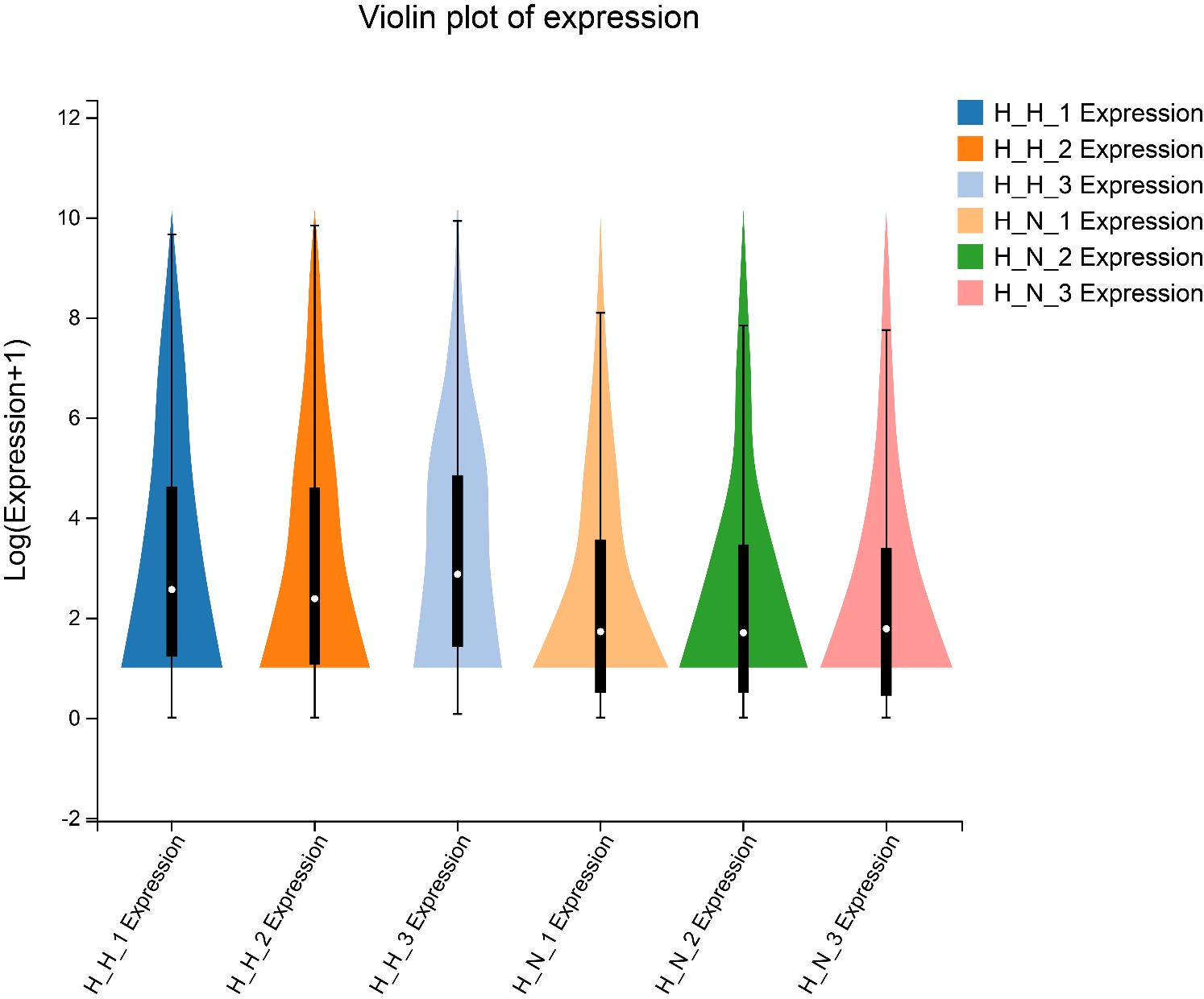


**Figure S5.** Violin plot of gene expression of DEGs for each sample of normoxia-treated healthy hPDLSCs (H_N) compared with hypoxia-treated healthy hPDLSCs (H_H). The width of the violin plot is proportional to the density of the data, the boxplots inside the violin plot indicate quantiles and outliers, and the white origin represents the median.


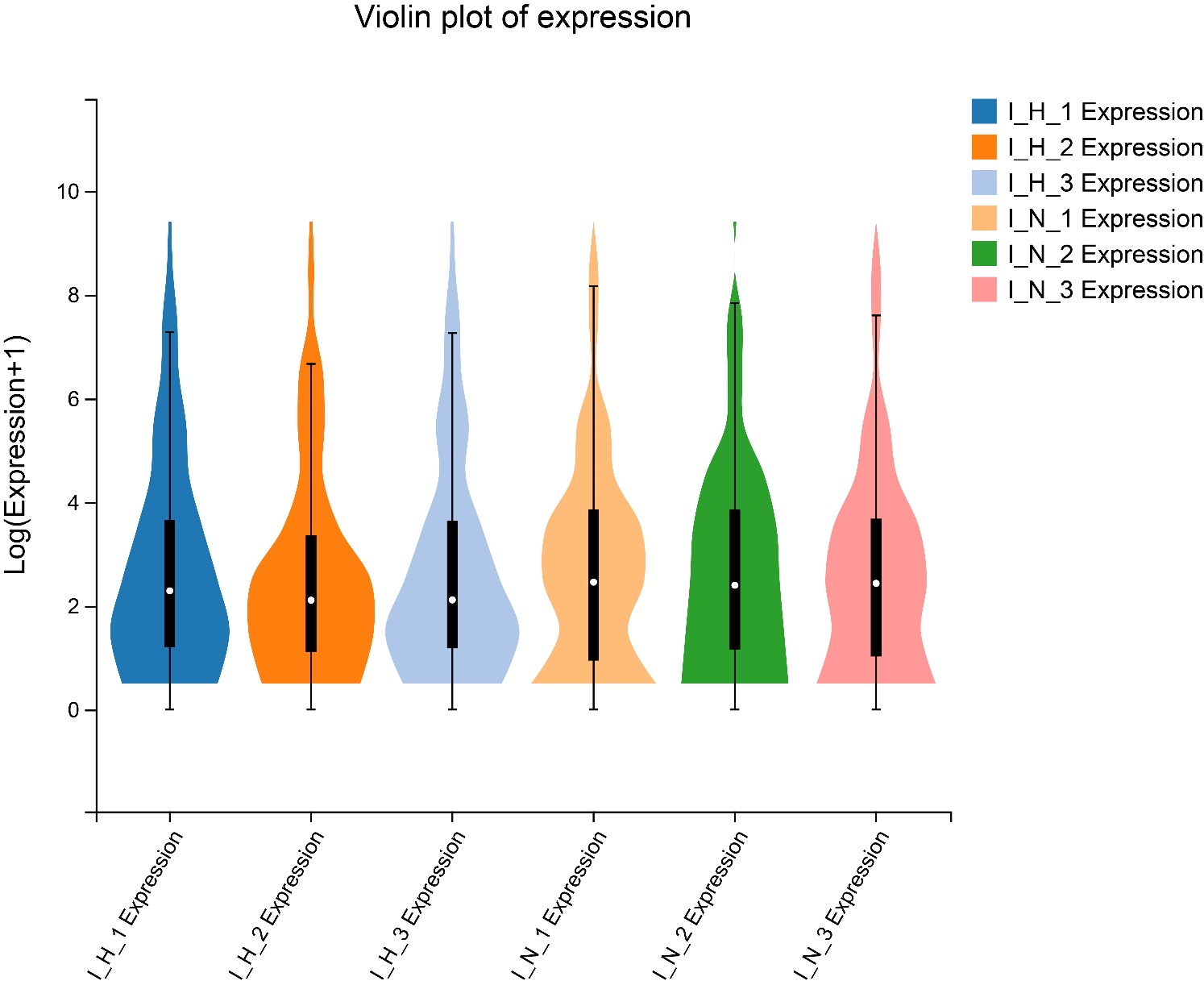


**Figure S6.** Violin plot of gene expression of DEGs for each sample of normoxia-treated inflammatory hPDLSCs (I_N) compared with hypoxia-treated inflammatory hPDLSCs (I_H). The width of the violin plot is proportional to the density of the data, the boxplots inside the violin plot indicate quantiles and outliers, and the white origin represents the median.


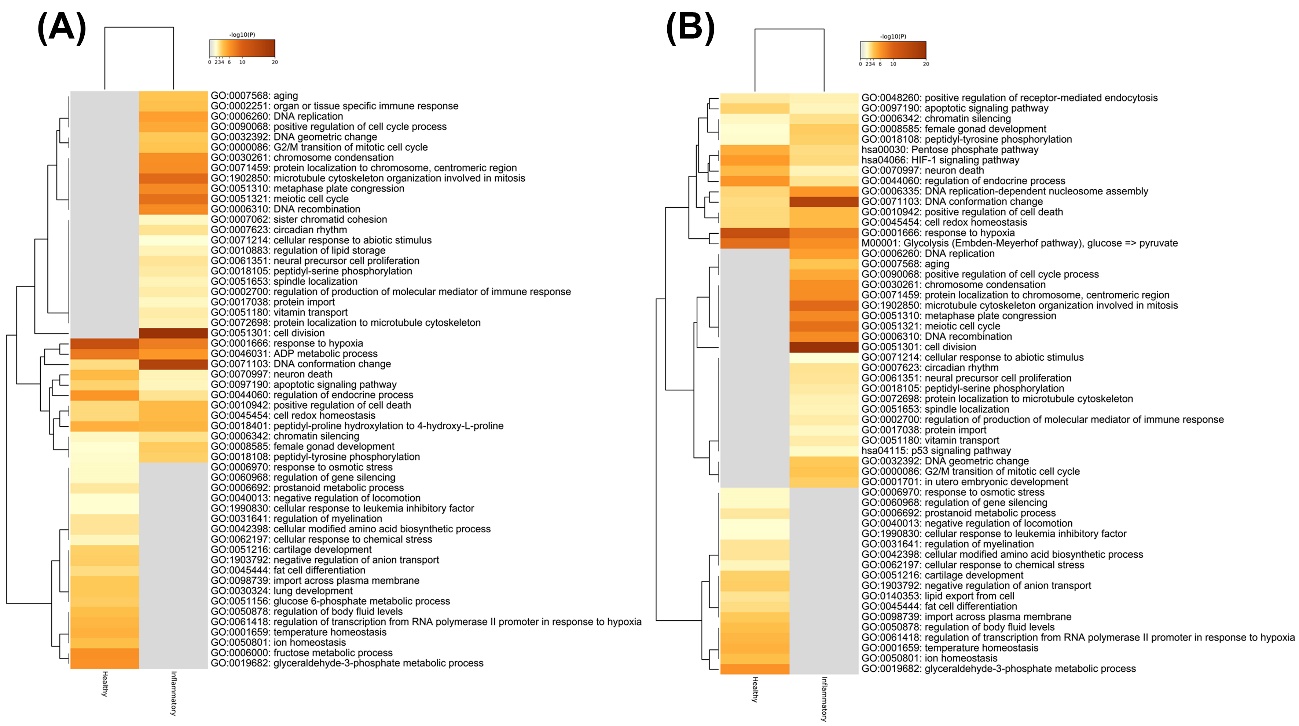


**Figure S7.** Heat map of the top 100 significantly enriched terms for DEGs in healthy hPDLSCs or inflammatory hPDLSCs. (A) Enrichment analysis based on GO biological processes. (B) Enrichment analysis based on GO biological processes and KEGG pathways. These enrichment analyses were conducted with the Metascape database and colored according to P value.


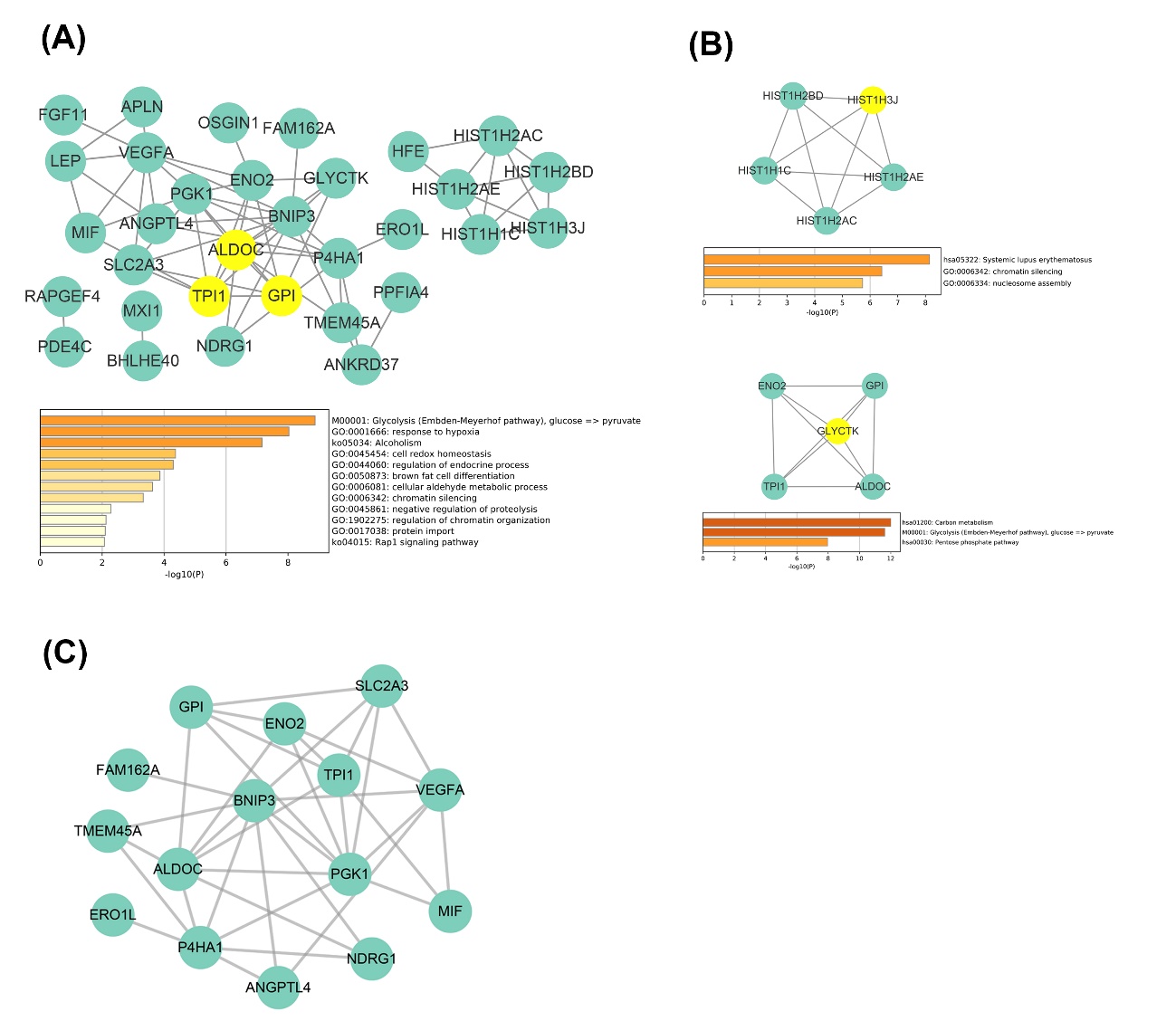


**Figure S8.** PPI network and clusters of DEGs in both healthy hPDLSCs and inflammatory hPDLSCs. (A) PPI network of DEGs. (B) Significant clusters identified by CytoHubba with the hub genes shown in yellow. (C) The 15 hub genes found among the common DEGs.


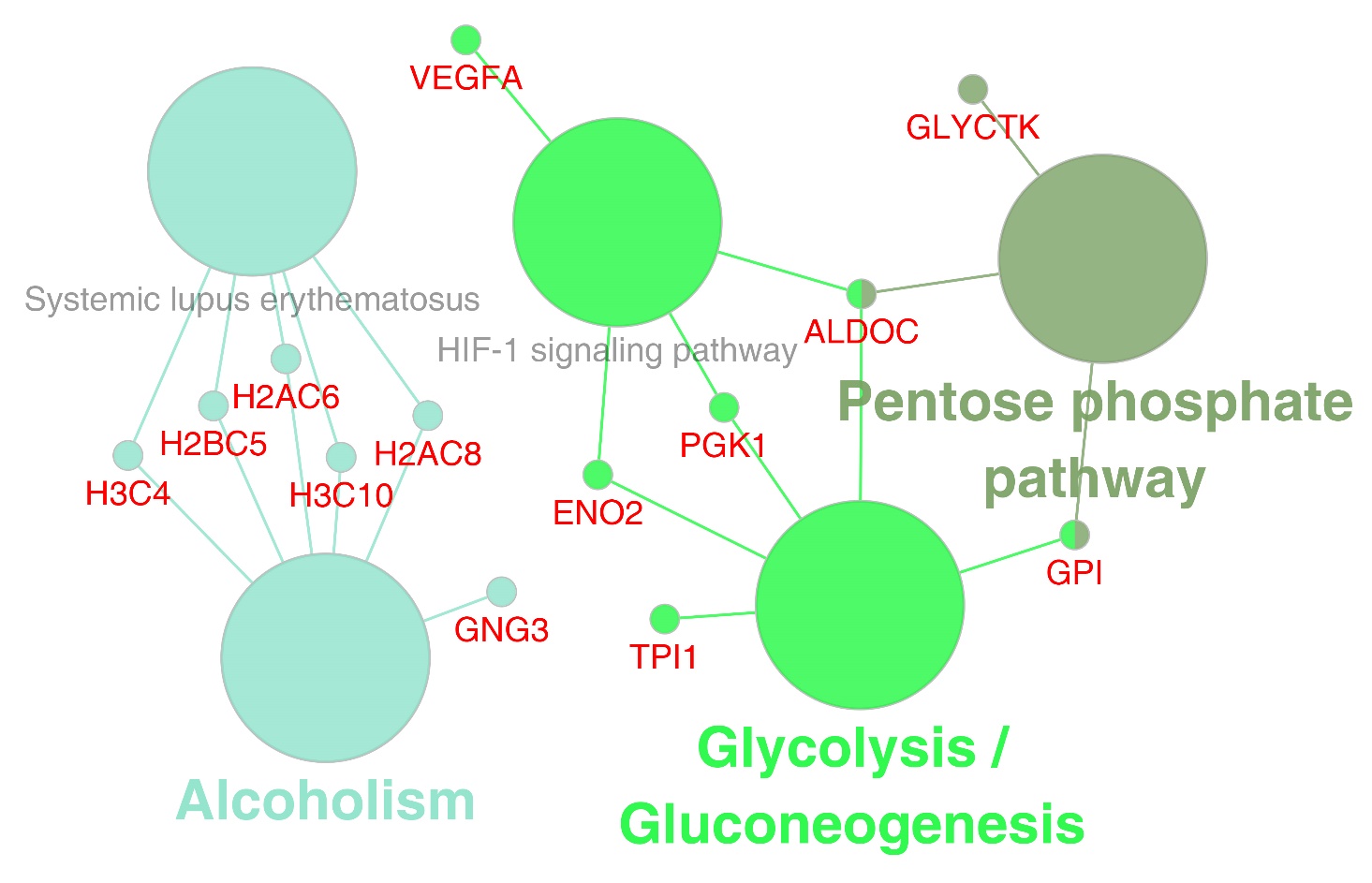


**Figure S9.** ClueGO KEGG pathway enrichment analysis of the DEGs in both healthy hPDLSCs and inflammatory hPDLSCs. Large circles are pathways. One color indicates a functional group. The sizes of circles depend on the P value. Pathways including three or more DEGs are presented. Small circles are genes indicated in the pathways. Genes expressed in more than two pathways are indicated by the fusion of the colors representing each pathway.

# Supplementary Tables

### Table S1. The 134 DEGs identified under the healthy condition

| **Gene ID** | **Gene Symbol** | **log2 (H_H / H_N)** | **P value** | **Q value** |
| --- | --- | --- | --- | --- |
| BGIG9606_60551 | 'BGIG9606_60551' | 20.59684853 | 1.44E-09 | 7.64E-07 |
| 112399 | 'EGLN3' | 6.814060887 | 2.45E-05 | 0.003369557 |
| 3952 | 'LEP' | 6.732358489 | 6.27E-06 | 0.001059582 |
| 2847 | 'MCHR1' | 6.673837677 | 5.03E-10 | 2.96E-07 |
| BGIG9606_68277 | 'BGIG9606_68277' | 6.13433626 | 3.31E-04 | 0.029232434 |
| 11000 | 'SLC27A3' | 6.100491982 | 1.36E-04 | 0.014155082 |
| 11069 | 'RAPGEF4' | 4.865509174 | 2.00E-05 | 0.002833132 |
| 8862 | 'APLN' | 4.21859594 | 1.07E-14 | 1.74E-11 |
| 5129 | 'CDK18' | 4.190680028 | 3.68E-07 | 1.02E-04 |
| 105373989 | 'LOC105373989' | 4.0843153 | 1.52E-07 | 4.96E-05 |
| 1959 | 'EGR2' | 3.746750435 | 1.54E-05 | 0.00233366 |
| 2256 | 'FGF11' | 3.611926811 | 4.64E-06 | 8.27E-04 |
| 257407 | 'C2orf72' | 3.543376837 | 2.48E-05 | 0.003369557 |
| 3012 | 'HIST1H2AE' | 3.49407099 | 1.57E-08 | 6.05E-06 |
| 3787 | 'KCNS1' | 3.34683459 | 1.66E-25 | 6.50E-22 |
| 8351 | 'HIST1H3D' | 3.325221431 | 2.58E-11 | 2.20E-08 |
| 9478 | 'CABP1' | 3.208603389 | 4.97E-04 | 0.040256871 |
| 23308 | 'ICOSLG' | 3.172733162 | 4.00E-10 | 2.53E-07 |
| 7472 | 'WNT2' | 3.09711938 | 1.70E-04 | 0.017051408 |
| 9241 | 'NOG' | 3.071397974 | 1.65E-06 | 3.52E-04 |
| 3625 | 'INHBB' | 2.980854988 | 1.77E-06 | 3.64E-04 |
| 280664 | 'WFDC10B' | 2.869711492 | 2.23E-07 | 6.82E-05 |
| 3017 | 'HIST1H2BD' | 2.84971632 | 3.52E-13 | 4.60E-10 |
| 8497 | 'PPFIA4' | 2.825701279 | 7.33E-24 | 2.40E-20 |
| 6781 | 'STC1' | 2.794995644 | 2.46E-18 | 5.35E-15 |
| 112268238 | 'LOC112268238' | 2.735383957 | 4.69E-13 | 5.74E-10 |
| 115330 | 'GPR146' | 2.713681373 | 7.30E-08 | 2.51E-05 |
| 5740 | 'PTGIS' | 2.6346245 | 1.23E-04 | 0.01300848 |
| 192668 | 'CYS1' | 2.620705836 | 9.24E-09 | 3.85E-06 |
| 8553 | 'BHLHE40' | 2.564088822 | 2.90E-07 | 8.62E-05 |
| 3486 | 'IGFBP3' | 2.524600763 | 6.72E-36 | 4.39E-32 |
| 205 | 'AK4' | 2.518944298 | 1.21E-25 | 5.93E-22 |
| BGIG9606_54780 | 'BGIG9606_54780' | 2.483437324 | 1.30E-08 | 5.18E-06 |
| 8347 | 'HIST1H2BC' | 2.457391142 | 3.82E-09 | 1.74E-06 |
| 230 | 'ALDOC' | 2.413522788 | 4.06E-11 | 3.32E-08 |
| 6676 | 'SPAG4' | 2.400124246 | 1.39E-05 | 0.002162593 |
| 8357 | 'HIST1H3H' | 2.358778908 | 6.59E-07 | 1.68E-04 |
| 8328 | 'GFI1B' | 2.279733721 | 1.20E-04 | 0.01296106 |
| 441054 | 'C4orf47' | 2.254294137 | 7.08E-10 | 3.85E-07 |
| 167359 | 'NIM1K' | 2.245822545 | 2.12E-04 | 0.020451399 |
| 5143 | 'PDE4C' | 2.245594341 | 2.18E-07 | 6.78E-05 |
| 4601 | 'MXI1' | 2.178502444 | 1.40E-08 | 5.49E-06 |
| 10397 | 'NDRG1' | 2.144356678 | 1.11E-12 | 1.28E-09 |
| 57091 | 'CASS4' | 2.14180713 | 2.68E-04 | 0.024383579 |
| 29923 | 'HILPDA' | 2.108988414 | 3.56E-07 | 1.01E-04 |
| 5603 | 'MAPK13' | 2.088331191 | 1.43E-07 | 4.74E-05 |
| 58494 | 'JAM2' | 2.051987919 | 2.22E-05 | 0.003105863 |
| BGIG9606_54773 | 'BGIG9606_54773' | 2.043791749 | 7.30E-05 | 0.008563799 |
| 5033 | 'P4HA1' | 2.032323388 | 1.05E-23 | 2.93E-20 |
| 3099 | 'HK2' | 2.028308802 | 1.26E-13 | 1.89E-10 |
| 8334 | 'HIST1H2AC' | 2.010433733 | 1.59E-06 | 3.50E-04 |
| 55304 | 'SPTLC3' | 1.978331795 | 8.04E-06 | 0.0013356 |
| 440689 | 'HIST2H2BF' | 1.973084011 | 2.52E-06 | 4.98E-04 |
| BGIG9606_49071 | 'BGIG9606_49071' | 1.941622594 | 6.00E-04 | 0.045745374 |
| 2026 | 'ENO2' | 1.924153507 | 3.80E-20 | 9.31E-17 |
| 55893 | 'ZNF395' | 1.902270676 | 1.91E-17 | 3.41E-14 |
| 7038 | 'TG' | 1.870780912 | 6.66E-04 | 0.049470271 |
| 285966 | 'TCAF2' | 1.860007037 | 4.68E-11 | 3.53E-08 |
| 664 | 'BNIP3' | 1.831541165 | 1.27E-42 | 1.24E-38 |
| 55076 | 'TMEM45A' | 1.812155149 | 2.17E-10 | 1.47E-07 |
| 7422 | 'VEGFA' | 1.798395243 | 3.09E-05 | 0.004121329 |
| 3006 | 'HIST1H1C' | 1.719091108 | 4.28E-05 | 0.005480831 |
| 54206 | 'ERRFI1' | 1.689177137 | 1.07E-06 | 2.52E-04 |
| BGIG9606_68545 | 'BGIG9606_68545' | 1.686170647 | 1.74E-05 | 0.002579633 |
| 1466 | 'CSRP2' | 1.632176611 | 7.33E-11 | 5.32E-08 |
| 5230 | 'PGK1' | 1.592337764 | 1.37E-13 | 1.92E-10 |
| 374887 | 'YJEFN3' | 1.565875095 | 2.41E-06 | 4.81E-04 |
| 26355 | 'FAM162A' | 1.562295655 | 8.56E-08 | 2.89E-05 |
| 56911 | 'MAP3K7CL' | 1.548519449 | 5.13E-04 | 0.040859821 |
| 79729 | 'SH3D21' | 1.538199713 | 1.55E-05 | 0.00233366 |
| 6515 | 'SLC2A3' | 1.507856253 | 1.49E-06 | 3.36E-04 |
| 114897 | 'C1QTNF1' | 1.495076716 | 5.14E-10 | 2.96E-07 |
| 353322 | 'ANKRD37' | 1.482326438 | 3.16E-09 | 1.51E-06 |
| 57232 | 'ZNF630' | 1.474134909 | 8.41E-05 | 0.009689195 |
| 10144 | 'FAM13A' | 1.442498915 | 4.37E-06 | 8.01E-04 |
| 7090 | 'TLE3' | 1.437037808 | 1.64E-06 | 3.52E-04 |
| 7045 | 'TGFBI' | 1.420825128 | 1.21E-05 | 0.001908444 |
| 2785 | 'GNG3' | 1.3997272 | 8.56E-07 | 2.07E-04 |
| 84033 | 'OBSCN' | 1.390651531 | 5.60E-07 | 1.46E-04 |
| 55876 | 'GSDMB' | 1.359960883 | 2.37E-09 | 1.19E-06 |
| 339105 | 'PRSS53' | 1.326250819 | 5.68E-06 | 9.76E-04 |
| 151295 | 'SLC23A3' | 1.321634223 | 4.94E-05 | 0.00616416 |
| 5210 | 'PFKFB4' | 1.266145534 | 3.61E-05 | 0.004694002 |
| 57561 | 'ARRDC3' | 1.25900571 | 3.83E-04 | 0.033216126 |
| 51129 | 'ANGPTL4' | 1.256939825 | 7.98E-18 | 1.56E-14 |
| 79844 | 'ZDHHC11' | 1.255993003 | 4.81E-05 | 0.006048361 |
| 5352 | 'PLOD2' | 1.251799566 | 1.61E-06 | 3.50E-04 |
| 3077 | 'HFE' | 1.233438224 | 4.69E-05 | 0.005962362 |
| BGIG9606_37544 | 'BGIG9606_37544' | 1.232734843 | 1.45E-05 | 0.002218146 |
| 25946 | 'ZNF385A' | 1.216758034 | 1.20E-04 | 0.01296106 |
| 143689 | 'PIWIL4' | 1.193253894 | 4.89E-04 | 0.039901516 |
| 571 | 'BACH1' | 1.182675981 | 7.81E-11 | 5.47E-08 |
| 388588 | 'SMIM1' | 1.181768682 | 4.70E-04 | 0.03887121 |
| 401152 | 'C4orf3' | 1.170164777 | 3.82E-07 | 1.04E-04 |
| 11346 | 'SYNPO' | 1.154795748 | 1.37E-12 | 1.49E-09 |
| 7167 | 'TPI1' | 1.151681131 | 2.10E-07 | 6.65E-05 |
| 5190 | 'PEX6' | 1.147971757 | 2.99E-07 | 8.74E-05 |
| 4282 | 'MIF' | 1.123262421 | 2.07E-12 | 2.13E-09 |
| 5209 | 'PFKFB3' | 1.117868304 | 4.18E-10 | 2.56E-07 |
| 78987 | 'CRELD1' | 1.111357516 | 1.69E-05 | 0.002532701 |
| 83882 | 'TSPAN10' | 1.105569738 | 1.85E-04 | 0.018441618 |
| 30001 | 'ERO1A' | 1.105384002 | 4.67E-11 | 3.53E-08 |
| 2821 | 'GPI' | 1.098325111 | 1.43E-06 | 3.26E-04 |
| 10500 | 'SEMA6C' | 1.078492899 | 7.64E-05 | 0.008912486 |
| 7442 | 'TRPV1' | 1.072628451 | 2.10E-08 | 7.90E-06 |
| 55818 | 'KDM3A' | 1.065826082 | 4.24E-12 | 4.15E-09 |
| 84074 | 'QRICH2' | 1.064709138 | 2.76E-04 | 0.025048915 |
| 79690 | 'GAL3ST4' | 1.057371951 | 1.63E-04 | 0.016759021 |
| 6385 | 'SDC4' | 1.044238442 | 8.51E-09 | 3.62E-06 |
| 84795 | 'PYROXD2' | 1.03800476 | 4.14E-08 | 1.45E-05 |
| 170384 | 'FUT11' | 1.037169014 | 7.93E-09 | 3.45E-06 |
| 9162 | 'DGKI' | 1.034758927 | 1.35E-05 | 0.002118492 |
| 83591 | 'THAP2' | 1.015565221 | 4.43E-06 | 8.04E-04 |
| 54979 | 'HRASLS2' | 1.006022659 | 6.15E-06 | 0.001048583 |
| 120103 | 'SLC36A4' | -1.005891662 | 3.21E-06 | 6.11E-04 |
| 7965 | 'AIMP2' | -1.024699801 | 8.14E-05 | 0.009442081 |
| 2730 | 'GCLM' | -1.027049401 | 1.10E-08 | 4.49E-06 |
| 10239 | 'AP3S2' | -1.059893332 | 4.44E-04 | 0.037302826 |
| 6888 | 'TALDO1' | -1.082806072 | 2.39E-08 | 8.84E-06 |
| 89978 | 'DPH6' | -1.12130931 | 1.08E-04 | 0.011990947 |
| 114112 | 'TXNRD3' | -1.211240159 | 9.25E-05 | 0.01047377 |
| 1646 | 'AKR1C2' | -1.244133521 | 5.62E-05 | 0.006793107 |
| 3091 | 'HIF1A' | -1.258841386 | 2.00E-04 | 0.019454244 |
| 100652748 | 'TIMM23B' | -1.282499295 | 4.09E-06 | 7.57E-04 |
| 29948 | 'OSGIN1' | -1.363412121 | 6.08E-10 | 3.40E-07 |
| 124935 | 'SLC43A2' | -1.380999671 | 6.35E-09 | 2.83E-06 |
| 1728 | 'NQO1' | -1.460363977 | 8.68E-12 | 8.10E-09 |
| 23657 | 'SLC7A11' | -1.487069069 | 1.20E-04 | 0.01296106 |
| 132158 | 'GLYCTK' | -1.698066997 | 2.24E-04 | 0.021326909 |
| 654 | 'BMP6' | -2.010578311 | 4.55E-04 | 0.038088382 |
| 5935 | 'RBM3' | -2.243506294 | 6.84E-70 | 1.34E-65 |
| 5073 | 'PARN' | -2.386344619 | 6.64E-04 | 0.049470271 |
| 2151 | 'F2RL2' | -2.388699571 | 1.75E-06 | 3.64E-04 |
| BGIG9606_67807 | 'BGIG9606_67807' | -7.694079624 | 1.66E-04 | 0.016927552 |

### Table S2. The top 20 significantly enriched GO terms under the healthy condition

| **GO_P Term Desc** | **GO_P Term Level2** | **Rich Ratio** | **P value** | **Q value** |
| --- | --- | --- | --- | --- |
| canonical glycolysis | metabolic process | 0.170731707 | 1.72E-09 | 1.99E-06 |
| cellular response to hypoxia | response to stimulus | 0.068965517 | 4.71E-09 | 2.73E-06 |
| gluconeogenesis | metabolic process | 0.090909091 | 1.25E-06 | 4.84E-04 |
| glycolytic process | metabolic process | 0.102040816 | 5.64E-06 | 0.001634959 |
| positive regulation of follicle-stimulating hormone secretion | regulation of biological process | 0.333333333 | 1.14E-05 | 0.00263255 |
| fructose metabolic process | metabolic process | 0.25 | 2.94E-05 | 0.003678058 |
| nucleosome assembly | cellular process | 0.041420118 | 3.03E-05 | 0.003678058 |
| regulation of signaling receptor activity | regulation of biological process | 0.024498886 | 2.41E-05 | 0.003678058 |
| cell differentiation | developmental process | 0.015477214 | 3.42E-05 | 0.003678058 |
| cellular response to leptin stimulus | response to stimulus | 0.230769231 | 3.81E-05 | 0.003678058 |
| negative regulation of hepatocyte growth factor biosynthetic process | metabolic process | 1 | 2.70E-05 | 0.003678058 |
| regulation of gene silencing | regulation of biological process | 0.230769231 | 3.81E-05 | 0.003678058 |
| regulation of transcription from RNA polymerase II promoter in response to hypoxia | metabolic process | 0.1 | 5.56E-05 | 0.004961299 |
| positive regulation of aldosterone secretion | signaling | 0.666666667 | 8.06E-05 | 0.006672968 |
| negative regulation of vasoconstriction | regulation of biological process | 0.5 | 1.61E-04 | 0.01163777 |
| positive regulation of ovulation | reproductive process | 0.5 | 1.61E-04 | 0.01163777 |
| DNA replication-dependent nucleosome assembly | cellular process | 0.136363636 | 1.98E-04 | 0.01350834 |
| response to hypoxia | response to stimulus | 0.03030303 | 2.14E-04 | 0.01380607 |
| fructose 2,6-bisphosphate metabolic process | metabolic process | 0.4 | 2.67E-04 | 0.01512098 |
| hemostasis | biological regulation | 0.066666667 | 2.74E-04 | 0.01512098 |

### Table S3. Significantly enriched GO terms in GSEA under the healthy condition

| **GO Term** | **NES** | **NOM p-val** | **FDR q-val** |
| --- | --- | --- | --- |
| ATP generation from ADP | 2.3804705 | 0 | 0 |
| ADP metabolic process | 2.3517394 | 0 | 0 |
| Glucose catabolic process | 2.302044 | 0 | 0 |
| Ribonucleoside diphosphate metabolic process | 2.2829602 | 0 | 0 |
| Nucleotide phosphorylation | 2.2111802 | 0 | 5.35E-04 |
| Hexose catabolic process | 2.1973605 | 0 | 5.94E-04 |
| NADH metabolic process | 2.154368 | 0 | 6.35E-04 |
| Nucleoside diphosphate metabolic process | 2.1239128 | 0 | 0.00122324 |
| Monosaccharide catabolic process | 2.077269 | 0 | 0.00237412 |
| Pyruvate metabolic process | 2.0346985 | 0 | 0.00471244 |
| NAD metabolic process | 2.03298 | 0 | 0.00444563 |
| Carbohydrate transmembrane transport | 1.9340992 | 0 | 0.02519618 |
| Regulation of t helper 1 type immune response | 1.9178443 | 0 | 0.03071443 |
| Antimicrobial humoral response | 1.9166893 | 0.00159236 | 0.02883775 |
| Protein hydroxylation | 1.8372083 | 0 | 0.08577249 |
| Negative regulation of adaptive immune response | 1.8361101 | 0 | 0.08141699 |
| Negative regulation of alpha beta t cell activation | 1.8327614 | 0 | 0.08002278 |
| Brown fat cell differentiation | 1.8157986 | 0.00163666 | 0.09505944 |
| Regulation of t helper cell differentiation | 1.7987231 | 0 | 0.11146784 |
| Cellular response to oxygen levels | 1.7948897 | 0 | 0.11144593 |
| Protein dealkylation | 1.7929772 | 0.00163934 | 0.10826278 |
| Positive regulation of organic acid transport | 1.7922182 | 0.00161551 | 0.10475585 |
| Innate immune response in mucosa | 1.7761984 | 0.00692042 | 0.12183497 |
| Regulation of icosanoid secretion | 1.7744273 | 0.00163934 | 0.11924081 |
| Decidualization | 1.7712942 | 0.00163666 | 0.11935511 |
| Negative regulation of cytokine production involved in immune response | 1.7684095 | 0.00510204 | 0.11900803 |
| Carbohydrate catabolic process | 1.7518295 | 0 | 0.14179465 |
| Neurotrophin TRK receptor signaling pathway | 1.7516354 | 0.00513699 | 0.1369222 |
| Regulation of cd4 positive alpha beta t cell activation | 1.7495451 | 0.0016835 | 0.13606821 |
| Excitatory postsynaptic potential | 1.7345794 | 0.00501672 | 0.15739958 |
| Maternal placenta development | 1.7310135 | 0.00314961 | 0.15843555 |
| Inorganic ion import into cell | 1.7260691 | 0.01282051 | 0.16214034 |
| Neuron apoptotic process | 1.7193714 | 0 | 0.17084853 |
| Regulation of organic acid transport | 1.7164849 | 0.00291971 | 0.17169487 |
| Negative regulation of cell aging | 1.7127423 | 0.00680272 | 0.1746218 |
| Glucose metabolic process | 1.7109888 | 0 | 0.17300689 |
| Regulation of calcium ion dependent exocytosis | 1.7023687 | 0.00142045 | 0.18558823 |
| Calcium ion regulated exocytosis of neurotransmitter | 1.6982386 | 0.01492537 | 0.18914127 |
| Regulation of insulin like growth factor receptor signaling pathway | 1.6903603 | 0.00166113 | 0.20091484 |
| Response to oxygen levels | 1.6881995 | 0 | 0.20037325 |
| Demethylation | 1.6823491 | 0.00151286 | 0.2087135 |
| Regulation of alpha beta t cell activation | 1.6803569 | 0.00144928 | 0.20803314 |
| Negative regulation of cd4 positive alpha beta t cell activation | 1.6783972 | 0.00848896 | 0.20738316 |
| Regulation of glucose metabolic process | 1.6747135 | 0 | 0.21021238 |
| Positive regulation of blood circulation | 1.6730919 | 0 | 0.20895651 |
| Positive regulation of heart contraction | 1.672243 | 0.00474684 | 0.20667607 |
| Import into cell | 1.6659217 | 0.00927357 | 0.21691926 |
| Response to fluid shear stress | 1.6654913 | 0.00806452 | 0.21326883 |
| Chromatin silencing | 1.6610895 | 0.00139276 | 0.2189095 |
| Collagen fibril organization | 1.6592709 | 0.01076923 | 0.21896408 |
| Neuron cell adhesion | 1.6546153 | 0.01196581 | 0.2253408 |
| Maternal process involved in female pregnancy | 1.6534057 | 0.00765697 | 0.22371116 |
| Neuron death | 1.6533495 | 0.00156986 | 0.21962462 |
| Regulation of sensory perception | 1.6526921 | 0.00651466 | 0.2171248 |
| Organ or tissue specific immune response | 1.6482359 | 0.0096463 | 0.22330822 |
| Steroid catabolic process | 1.6442103 | 0.00838926 | 0.22839504 |
| Regulation of positive chemotaxis | 1.6440699 | 0.00504202 | 0.22465336 |
| Positive regulation of t cell mediated cytotoxicity | 1.641479 | 0.01541096 | 0.22628762 |
| Protein refolding | 1.6397935 | 0.01954397 | 0.22585946 |
| Phosphatidylcholine acyl chain remodeling | 1.6389141 | 0.01176471 | 0.22411238 |
| Regulation of cytokine production involved in immune response | 1.6364179 | 0.00773994 | 0.22576925 |
| Negative regulation of organ growth | 1.6262413 | 0.02208202 | 0.2457903 |

### Table S4. The top 20 significantly enriched KEGG pathways under the healthy condition

| **KEGG Pathway Term Desc** | **KEGG Pathway Term Level1** | **Rich Ratio** | **P value** | **Q value** |
| --- | --- | --- | --- | --- |
| Glycolysis / Gluconeogenesis | Metabolism | 0.074468085 | 2.55E-06 | 3.64E-04 |
| Carbon metabolism | Metabolism | 0.047619048 | 3.82E-06 | 3.64E-04 |
| Pentose phosphate pathway | Metabolism | 0.12195122 | 6.50E-06 | 3.97E-04 |
| Alcoholism | Human Diseases | 0.043269231 | 8.31E-06 | 3.97E-04 |
| Fructose and mannose metabolism | Metabolism | 0.106382979 | 1.29E-05 | 4.46E-04 |
| Systemic lupus erythematosus | Human Diseases | 0.040540541 | 1.40E-05 | 4.46E-04 |
| Microbial metabolism in diverse environments | Metabolism | 0.036 | 3.58E-05 | 9.77E-04 |
| Biosynthesis of antibiotics | Metabolism | 0.03058104 | 5.21E-05 | 0.001242953 |
| Biosynthesis of secondary metabolites | Metabolism | 0.023297491 | 6.44E-05 | 0.001367437 |
| HIF-1 signaling pathway | Environmental Information Processing | 0.040540541 | 4.04E-04 | 0.00771985 |
| Biosynthesis of amino acids | Metabolism | 0.048076923 | 5.78E-04 | 0.010028424 |
| Methane metabolism | Metabolism | 0.083333333 | 0.001622554 | 0.025825651 |
| Ubiquinone and other terpenoid-quinone biosynthesis | Metabolism | 0.153846154 | 0.003105564 | 0.042368766 |
| Carbon fixation in photosynthetic organisms | Metabolism | 0.068181818 | 0.002898835 | 0.042368766 |
| Starch and sucrose metabolism | Metabolism | 0.058823529 | 0.004411236 | 0.056169738 |
| TGF-beta signaling pathway | Environmental Information Processing | 0.036363636 | 0.005710489 | 0.068168962 |
| Amino sugar and nucleotide sugar metabolism | Metabolism | 0.046875 | 0.008305919 | 0.093319443 |
| AMPK signaling pathway | Environmental Information Processing | 0.022857143 | 0.02718895 | 0.273320497 |
| Renal cell carcinoma | Human Diseases | 0.030612245 | 0.02590105 | 0.273320497 |
| Neomycin, kanamycin and gentamicin biosynthesis | Metabolism | 0.166666667 | 0.03832093 | 0.31014114 |

### Table S5. Significantly enriched KEGG pathways in GSEA under the healthy condition

| **KEGG Term Desc** | **NES** | **NOM p-val** | **FDR q-val** |
| --- | --- | --- | --- |
| Glycolysis / Gluconeogenesis | 1.9702411 | 0 | 0.011256628 |
| Systemic lupus erythematosus | 1.8309753 | 0 | 0.02951324 |
| Fructose and mannose metabolism | 1.8269514 | 0.00286123 | 0.020432202 |
| Pentose phosphate pathway | 1.7452494 | 0.00739645 | 0.04210581 |
| ECM-receptor interaction | 1.5841103 | 0.002624672 | 0.17592944 |
| Galactose metabolism | 1.5433843 | 0.030395137 | 0.2094142 |
| Arrhythmogenic right ventricular cardiomyopathy (ARVC) | 1.5141789 | 0.009198424 | 0.23111723 |
| Complement and coagulation cascades | 1.5045204 | 0.010796221 | 0.21990642 |
| Primary immunodeficiency | 1.5019447 | 0.04117647 | 0.20044191 |
| Notch signaling pathway | 1.4684031 | 0.0261708 | 0.23960765 |

### Table S6. The 164 DEGs identified under the inflammatory condition

| **Gene ID** | **Gene Symbol** | **log2 (I_H / I_N)** | **P value** | **Q value** |
| --- | --- | --- | --- | --- |
| BGIG9606_68131 | 'BGIG9606_68131' | 6.989819543 | 4.07E-06 | 7.08E-04 |
| BGIG9606_54777 | 'BGIG9606_54777' | 5.251430378 | 4.17E-04 | 0.024673634 |
| 3952 | 'LEP' | 4.7511114 | 6.66E-05 | 0.006003947 |
| 8343 | 'HIST1H2BF' | 4.070424015 | 8.99E-08 | 3.10E-05 |
| 11069 | 'RAPGEF4' | 3.951212155 | 2.19E-05 | 0.002604488 |
| BGIG9606_36518 | 'BGIG9606_36518' | 3.715037679 | 6.33E-04 | 0.0331751 |
| 8351 | 'HIST1H3D' | 3.479364708 | 3.12E-16 | 1.21E-12 |
| 3012 | 'HIST1H2AE' | 3.353548115 | 1.98E-08 | 9.79E-06 |
| 5129 | 'CDK18' | 3.015957636 | 1.50E-05 | 0.00188391 |
| 2256 | 'FGF11' | 2.92840027 | 2.90E-06 | 5.49E-04 |
| 3017 | 'HIST1H2BD' | 2.906410981 | 1.51E-07 | 4.79E-05 |
| 115330 | 'GPR146' | 2.789508819 | 6.95E-07 | 1.66E-04 |
| BGIG9606_43501 | 'BGIG9606_43501' | 2.775494605 | 3.89E-04 | 0.023373397 |
| 105373989 | 'LOC105373989' | 2.6510477 | 6.74E-04 | 0.034675079 |
| BGIG9606_68386 | 'BGIG9606_68386' | 2.512762537 | 1.64E-04 | 0.011904242 |
| 8862 | 'APLN' | 2.489701307 | 3.01E-04 | 0.018826784 |
| 8497 | 'PPFIA4' | 2.324760212 | 4.09E-08 | 1.75E-05 |
| BGIG9606_54780 | 'BGIG9606_54780' | 2.230874851 | 1.47E-04 | 0.011000583 |
| 6676 | 'SPAG4' | 2.221323717 | 3.96E-06 | 6.94E-04 |
| 112268238 | 'LOC112268238' | 2.15893475 | 1.83E-06 | 3.75E-04 |
| 3625 | 'INHBB' | 2.110563067 | 5.96E-04 | 0.031871709 |
| 8553 | 'BHLHE40' | 2.039926739 | 8.99E-04 | 0.042808723 |
| 4601 | 'MXI1' | 1.983929024 | 2.46E-22 | 1.58E-18 |
| 5143 | 'PDE4C' | 1.975753911 | 8.72E-05 | 0.007348068 |
| 8334 | 'HIST1H2AC' | 1.971674263 | 3.15E-09 | 2.14E-06 |
| 55803 | 'ADAP2' | 1.88590044 | 0.001077178 | 0.049011619 |
| 10397 | 'NDRG1' | 1.855994993 | 5.65E-06 | 9.08E-04 |
| 230 | 'ALDOC' | 1.804281271 | 4.99E-14 | 1.07E-10 |
| 441054 | 'C4orf47' | 1.798883364 | 1.35E-09 | 1.04E-06 |
| 285966 | 'TCAF2' | 1.794040131 | 5.07E-06 | 8.38E-04 |
| 8357 | 'HIST1H3H' | 1.769517008 | 5.29E-04 | 0.029136207 |
| 280664 | 'WFDC10B' | 1.735462697 | 2.88E-04 | 0.018299195 |
| 6285 | 'S100B' | 1.691698576 | 5.41E-06 | 8.77E-04 |
| 5745 | 'PTH1R' | 1.683605522 | 1.31E-05 | 0.00173154 |
| 55893 | 'ZNF395' | 1.676443144 | 7.46E-05 | 0.006421763 |
| 5033 | 'P4HA1' | 1.585723615 | 2.11E-09 | 1.51E-06 |
| 8349 | 'HIST2H2BE' | 1.566414974 | 2.59E-05 | 0.002937815 |
| 2026 | 'ENO2' | 1.543140121 | 1.60E-08 | 8.44E-06 |
| 664 | 'BNIP3' | 1.537798746 | 8.82E-09 | 5.01E-06 |
| 55076 | 'TMEM45A' | 1.525546674 | 7.84E-16 | 2.52E-12 |
| 7422 | 'VEGFA' | 1.524210155 | 2.91E-16 | 1.21E-12 |
| 26355 | 'FAM162A' | 1.492436652 | 1.91E-04 | 0.013580162 |
| 55876 | 'GSDMB' | 1.483693478 | 1.95E-10 | 2.35E-07 |
| 3006 | 'HIST1H1C' | 1.453910238 | 6.84E-08 | 2.54E-05 |
| 353322 | 'ANKRD37' | 1.442775967 | 6.18E-10 | 5.67E-07 |
| 83538 | 'TTC25' | 1.43881201 | 8.66E-04 | 0.041765429 |
| 79729 | 'SH3D21' | 1.405900845 | 2.84E-28 | 2.74E-24 |
| 5230 | 'PGK1' | 1.357219241 | 1.62E-08 | 8.44E-06 |
| 3718 | 'JAK3' | 1.333823564 | 5.42E-05 | 0.005154109 |
| 54541 | 'DDIT4' | 1.330543508 | 2.76E-05 | 0.003061532 |
| 29923 | 'HILPDA' | 1.323284078 | 2.91E-04 | 0.01843049 |
| 6513 | 'SLC2A1' | 1.300220883 | 6.55E-06 | 0.001035142 |
| 84171 | 'LOXL4' | 1.285524571 | 2.30E-05 | 0.002678637 |
| 2785 | 'GNG3' | 1.279425265 | 3.26E-05 | 0.003508865 |
| 388389 | 'CCDC103' | 1.274360099 | 0.001030468 | 0.047164552 |
| 5190 | 'PEX6' | 1.249432504 | 1.98E-05 | 0.002385992 |
| 83882 | 'TSPAN10' | 1.244030675 | 5.56E-04 | 0.030319954 |
| 6515 | 'SLC2A3' | 1.240578888 | 3.53E-10 | 3.78E-07 |
| 148523 | 'CIART' | 1.21467156 | 1.83E-05 | 0.002243005 |
| 949 | 'SCARB1' | 1.203537762 | 1.20E-05 | 0.001620524 |
| 3077 | 'HFE' | 1.196174983 | 0.001031694 | 0.047164552 |
| BGIG9606_37544 | 'BGIG9606_37544' | 1.182307011 | 1.45E-05 | 0.001835886 |
| 85236 | 'HIST1H2BK' | 1.177380926 | 1.16E-05 | 0.001590499 |
| 151295 | 'SLC23A3' | 1.175252974 | 2.29E-08 | 1.11E-05 |
| 170384 | 'FUT11' | 1.165733497 | 3.59E-07 | 9.61E-05 |
| 55818 | 'KDM3A' | 1.150208855 | 7.13E-11 | 9.82E-08 |
| 54583 | 'EGLN1' | 1.14867329 | 1.82E-08 | 9.24E-06 |
| 81794 | 'ADAMTS10' | 1.147207704 | 6.60E-04 | 0.034061719 |
| 4783 | 'NFIL3' | 1.127678648 | 2.64E-04 | 0.017297553 |
| 388591 | 'RNF207' | 1.118311996 | 8.68E-04 | 0.041765429 |
| 2821 | 'GPI' | 1.103871643 | 5.05E-05 | 0.004898051 |
| 4282 | 'MIF' | 1.101006166 | 6.43E-08 | 2.48E-05 |
| 51129 | 'ANGPTL4' | 1.091933857 | 4.58E-10 | 4.42E-07 |
| 201232 | 'SLC16A13' | 1.077876912 | 0.001010393 | 0.046632791 |
| 196403 | 'DTX3' | 1.059223531 | 1.04E-06 | 2.33E-04 |
| 339105 | 'PRSS53' | 1.045789245 | 1.38E-04 | 0.01050843 |
| 7593 | 'MZF1' | 1.043594131 | 5.98E-04 | 0.031872975 |
| 83719 | 'YPEL3' | 1.021662266 | 9.80E-04 | 0.045760615 |
| BGIG9606_55385 | 'BGIG9606_55385' | 1.018013385 | 1.26E-04 | 0.009814632 |
| 8372 | 'HYAL3' | 1.01643202 | 3.79E-05 | 0.003944836 |
| 30001 | 'ERO1A' | 1.008304234 | 6.32E-08 | 2.48E-05 |
| 7167 | 'TPI1' | 1.006376972 | 4.72E-06 | 7.92E-04 |
| 4751 | 'NEK2' | -1.001727366 | 6.45E-05 | 0.005925147 |
| 55215 | 'FANCI' | -1.002249647 | 4.23E-06 | 7.28E-04 |
| 55355 | 'HJURP' | -1.003361986 | 1.18E-04 | 0.009228906 |
| 11277 | 'TREX1' | -1.006863661 | 6.09E-04 | 0.03227882 |
| 9319 | 'TRIP13' | -1.007576586 | 1.04E-05 | 0.001495079 |
| 1063 | 'CENPF' | -1.008703772 | 7.25E-07 | 1.71E-04 |
| 3838 | 'KPNA2' | -1.009426601 | 2.50E-06 | 4.92E-04 |
| 374383 | 'NCR3LG1' | -1.019130451 | 8.56E-07 | 1.99E-04 |
| 9928 | 'KIF14' | -1.025231419 | 2.09E-06 | 4.21E-04 |
| 54443 | 'ANLN' | -1.032065348 | 1.10E-05 | 0.001542817 |
| 8208 | 'CHAF1B' | -1.035165054 | 9.80E-04 | 0.045760615 |
| 3070 | 'HELLS' | -1.040517605 | 6.86E-04 | 0.034987233 |
| 9232 | 'PTTG1' | -1.042000935 | 4.46E-08 | 1.87E-05 |
| 9088 | 'PKMYT1' | -1.042846133 | 1.98E-04 | 0.013805383 |
| 9493 | 'KIF23' | -1.056657057 | 8.45E-08 | 3.02E-05 |
| 145508 | 'CEP128' | -1.072463198 | 6.87E-04 | 0.034987233 |
| 10196 | 'PRMT3' | -1.082233304 | 7.37E-08 | 2.68E-05 |
| 2237 | 'FEN1' | -1.083627897 | 2.83E-04 | 0.01807796 |
| 7153 | 'TOP2A' | -1.090464493 | 3.90E-05 | 0.003977194 |
| 4001 | 'LMNB1' | -1.090824896 | 9.95E-04 | 0.046246754 |
| 54892 | 'NCAPG2' | -1.091442103 | 8.52E-10 | 7.18E-07 |
| 54478 | 'PIMREG' | -1.096134219 | 9.97E-04 | 0.046246754 |
| 54821 | 'ERCC6L' | -1.101399878 | 6.23E-05 | 0.005750168 |
| 100652748 | 'TIMM23B' | -1.114421478 | 4.77E-04 | 0.027144276 |
| 1062 | 'CENPE' | -1.116975398 | 8.83E-09 | 5.01E-06 |
| 79801 | 'SHCBP1' | -1.118256132 | 8.43E-06 | 0.001270498 |
| 39 | 'ACAT2' | -1.118649759 | 3.43E-08 | 1.50E-05 |
| 11130 | 'ZWINT' | -1.127450241 | 1.25E-05 | 0.001657287 |
| 7272 | 'TTK' | -1.132476519 | 1.49E-05 | 0.00188391 |
| 81610 | 'FAM83D' | -1.14343342 | 6.30E-09 | 3.92E-06 |
| 7296 | 'TXNRD1' | -1.144579687 | 3.36E-08 | 1.50E-05 |
| 11004 | 'KIF2C' | -1.145896905 | 3.09E-05 | 0.00337072 |
| 7443 | 'VRK1' | -1.146596394 | 2.65E-04 | 0.017297553 |
| 3149 | 'HMGB3' | -1.160063192 | 4.43E-15 | 1.07E-11 |
| 699 | 'BUB1' | -1.162959955 | 9.46E-07 | 2.15E-04 |
| 374393 | 'FAM111B' | -1.164184622 | 1.89E-04 | 0.013468911 |
| 9833 | 'MELK' | -1.17642846 | 3.52E-10 | 3.78E-07 |
| 57082 | 'KNL1' | -1.177881957 | 5.05E-08 | 2.07E-05 |
| 51203 | 'NUSAP1' | -1.185787985 | 7.86E-05 | 0.006651442 |
| 64946 | 'CENPH' | -1.195053342 | 3.86E-05 | 0.003977194 |
| 983 | 'CDK1' | -1.195824435 | 2.15E-04 | 0.014739837 |
| 84296 | 'GINS4' | -1.198567914 | 1.36E-05 | 0.001766869 |
| 3832 | 'KIF11' | -1.200516437 | 3.13E-06 | 5.76E-04 |
| 55872 | 'PBK' | -1.202215371 | 3.22E-09 | 2.14E-06 |
| 63967 | 'CLSPN' | -1.221540765 | 0.001084014 | 0.049206568 |
| 259266 | 'ASPM' | -1.22281263 | 2.71E-04 | 0.017515618 |
| 9585 | 'KIF20B' | -1.224015683 | 1.52E-07 | 4.79E-05 |
| 195828 | 'ZNF367' | -1.247915017 | 2.25E-05 | 0.002649537 |
| 29948 | 'OSGIN1' | -1.248943195 | 2.16E-11 | 3.47E-08 |
| 3833 | 'KIFC1' | -1.25454041 | 2.95E-04 | 0.018628231 |
| 157313 | 'CDCA2' | -1.254971044 | 8.88E-08 | 3.10E-05 |
| 6241 | 'RRM2' | -1.257453919 | 5.42E-12 | 1.05E-08 |
| 675 | 'BRCA2' | -1.275134467 | 8.85E-07 | 2.03E-04 |
| 7804 | 'LRP8' | -1.276483867 | 3.69E-04 | 0.022357108 |
| 90835 | 'CCDC189' | -1.296287022 | 4.59E-04 | 0.026512207 |
| 80071 | 'CCDC15' | -1.302521626 | 1.00E-04 | 0.00809491 |
| 10403 | 'NDC80' | -1.306756481 | 2.48E-07 | 6.94E-05 |
| 114112 | 'TXNRD3' | -1.318351306 | 2.34E-05 | 0.00270301 |
| 23397 | 'NCAPH' | -1.321823077 | 1.84E-07 | 5.56E-05 |
| 22989 | 'MYH15' | -1.325993532 | 3.24E-05 | 0.003508865 |
| 10635 | 'RAD51AP1' | -1.344911998 | 4.77E-07 | 1.21E-04 |
| 1728 | 'NQO1' | -1.356128885 | 6.96E-09 | 4.20E-06 |
| 701 | 'BUB1B' | -1.36120318 | 1.92E-07 | 5.70E-05 |
| 57405 | 'SPC25' | -1.384298743 | 3.88E-06 | 6.86E-04 |
| 220134 | 'SKA1' | -1.425203665 | 4.29E-07 | 1.10E-04 |
| 9134 | 'CCNE2' | -1.459915488 | 6.35E-04 | 0.0331751 |
| 4605 | 'MYBL2' | -1.469769128 | 5.80E-04 | 0.031239989 |
| 5983 | 'RFC3' | -1.480421594 | 2.68E-07 | 7.29E-05 |
| 147841 | 'SPC24' | -1.496729338 | 3.15E-06 | 5.76E-04 |
| 55723 | 'ASF1B' | -1.598772729 | 1.45E-04 | 0.010907514 |
| 9156 | 'EXO1' | -1.620636291 | 7.25E-05 | 0.006321049 |
| 132158 | 'GLYCTK' | -1.674678387 | 1.16E-05 | 0.001590499 |
| 284992 | 'CCDC150' | -1.733949295 | 2.07E-04 | 0.014302926 |
| 118460 | 'EXOSC6' | -1.788938071 | 5.58E-05 | 0.005277419 |
| 5935 | 'RBM3' | -2.221108612 | 3.42E-62 | 6.60E-58 |
| 5739 | 'PTGIR' | -2.338100427 | 4.13E-04 | 0.024535748 |
| 25855 | 'BRMS1' | -3.359501576 | 2.75E-08 | 1.30E-05 |
| BGIG9606_55304 | 'BGIG9606_55304' | -5.052731617 | 1.02E-05 | 0.001481271 |
| BGIG9606_52562 | 'BGIG9606_52562' | -6.449158393 | 1.19E-05 | 0.001611106 |
| BGIG9606_49202 | 'BGIG9606_49202' | -6.823311557 | 2.69E-06 | 5.19E-04 |
| 222659 | 'PXT1' | -8.281381972 | 8.56E-10 | 7.18E-07 |
| 207063 | 'DHRSX' | -21.66983223 | 1.03E-09 | 8.27E-07 |

### Table S7. The top 20 significantly enriched GO terms under the inflammatory condition

| **GO_P Term Desc** | **GO_P Term Level2** | **Rich Ratio** | **P value** | **Q value** |
| --- | --- | --- | --- | --- |
| cell cycle | cellular process | 0.04881266 | 4.42E-22 | 5.27E-19 |
| cell division | cellular process | 0.05308219 | 1.43E-19 | 8.53E-17 |
| chromosome segregation | cellular process | 0.11320755 | 5.09E-12 | 2.02E-09 |
| mitotic cell cycle | cellular process | 0.04924242 | 2.09E-08 | 6.23E-06 |
| metaphase plate congression | localization | 0.3125 | 4.58E-08 | 1.09E-05 |
| nucleosome assembly | cellular process | 0.0591716 | 1.73E-07 | 3.44E-05 |
| DNA replication-dependent nucleosome assembly | cellular component organization or biogenesis | 0.22727273 | 2.68E-07 | 4.56E-05 |
| chromosome condensation | cellular process | 0.17241379 | 1.16E-06 | 1.73E-04 |
| mitotic spindle assembly checkpoint | regulation of biological process | 0.14705882 | 2.65E-06 | 3.52E-04 |
| DNA replication | metabolic process | 0.04761905 | 4.40E-06 | 5.24E-04 |
| cellular response to hypoxia | response to stimulus | 0.05517241 | 5.15E-06 | 5.59E-04 |
| microtubule-based movement | cellular process | 0.06603774 | 6.27E-06 | 6.22E-04 |
| canonical glycolysis | metabolic process | 0.12195122 | 6.89E-06 | 6.32E-04 |
| mitotic sister chromatid segregation | cellular process | 0.11904762 | 7.78E-06 | 6.62E-04 |
| mitotic spindle organization | cellular process | 0.1 | 1.86E-05 | 0.0014765 |
| positive regulation of follicle-stimulating hormone secretion | regulation of biological process | 0.33333333 | 2.23E-05 | 0.0016585 |
| negative regulation of hepatocyte growth factor biosynthetic process | metabolic process | 1 | 4.23E-05 | 0.00286809 |
| attachment of mitotic spindle microtubules to kinetochore | localization | 0.27272727 | 4.33E-05 | 0.00286809 |
| gluconeogenesis | metabolic process | 0.07575758 | 7.21E-05 | 0.00402859 |
| protein localization to kinetochore | localization | 0.23076923 | 7.44E-05 | 0.00402859 |

### Table S8. Significantly enriched GO terms in GSEA under the inflammatory condition

| **GO Term** | **NES** | **NOM p-val** | **FDR q-val** |
| --- | --- | --- | --- |
| Glucose Catabolic Process | 2.2431312 | 0 | 0.0050199 |
| ATP Generation from ADP | 2.16528 | 0 | 0.0090198 |
| ADP Metabolic Process | 2.125426 | 0 | 0.009346 |
| Ribonucleoside Diphosphate Metabolic Process | 2.1041806 | 0 | 0.0102603 |
| Negative Regulation of Cytokine Production Involved in Immune Response | 2.0557246 | 0 | 0.0218517 |
| NADH Metabolic Process | 2.0095685 | 0 | 0.036009 |
| Positive Regulation of Cell Matrix Adhesion | 1.9735152 | 0 | 0.0504239 |
| Pyruvate Metabolic Process | 1.93433 | 0 | 0.0752227 |
| Monosaccharide Catabolic Process | 1.9292784 | 0 | 0.0722983 |
| Hexose Catabolic Process | 1.9260656 | 0 | 0.0677496 |
| Nucleotide Phosphorylation | 1.9193101 | 0 | 0.0663984 |
| Blood Vessel Endothelial Cell Migration | 1.9073147 | 0 | 0.0724279 |
| NAD Metabolic Process | 1.9032577 | 0 | 0.0694688 |
| Polyol Transport | 1.8727977 | 0.0019048 | 0.0950583 |
| Organ or Tissue Specific Immune Response | 1.8514004 | 0 | 0.1165255 |
| Protein Hydroxylation | 1.8510908 | 0.0018315 | 0.1094933 |
| Regulation of Striated Muscle Cell Apoptotic Process | 1.8164929 | 0.0036765 | 0.1548836 |
| Positive Regulation of Vascular Endothelial Growth Factor Receptor Signaling Pathway | 1.793794 | 0 | 0.1893161 |
| Regulation Of Cellular Extravasation | 1.7861202 | 0.0036563 | 0.1971974 |
| Acylglycerol Homeostasis | 1.777974 | 0.0056818 | 0.2049431 |
| Endothelial Cell Migration | 1.7703948 | 0.0017637 | 0.2103844 |
| Axonemal Dynein Complex Assembly | 1.7688386 | 0.005814 | 0.2043355 |
| Carbohydrate Transmembrane Transport | 1.7634541 | 0.0055659 | 0.2069227 |
| Sensory Perception of Temperature Stimulus | 1.7563289 | 0.0037594 | 0.2133323 |
| Brown Fat Cell Differentiation | 1.7551434 | 0.0036969 | 0.2072736 |
| Antimicrobial Humoral Response | 1.7539146 | 0.0036832 | 0.2025187 |

### Table S9. The top 20 significantly enriched KEGG pathways under the inflammatory condition

| **KEGG Pathway Term Desc** | **KEGG Pathway Term Level1** | **Rich Ratio** | **P value** | **Q value** |
| --- | --- | --- | --- | --- |
| Alcoholism | Human Diseases | 0.04807692 | 1.29E-06 | 2.26E-04 |
| Systemic lupus erythematosus | Human Diseases | 0.04054054 | 1.77E-05 | 0.00155631 |
| Cell cycle | Cellular Processes | 0.03910615 | 1.97E-04 | 0.0115465 |
| Carbon metabolism | Metabolism | 0.03703704 | 2.74E-04 | 0.01207605 |
| Glycolysis / Gluconeogenesis | Metabolism | 0.05319149 | 4.14E-04 | 0.0145795 |
| Microbial metabolism in diverse environments | Metabolism | 0.028 | 0.00143582 | 0.03439766 |
| Biosynthesis of antibiotics | Metabolism | 0.02446483 | 0.00156353 | 0.03439766 |
| Viral carcinogenesis | Human Diseases | 0.025 | 0.00136365 | 0.03439766 |
| Methane metabolism | Metabolism | 0.08333333 | 0.00176176 | 0.0344522 |
| Pentose phosphate pathway | Metabolism | 0.07317073 | 0.00256752 | 0.04448013 |
| Ubiquinone and other terpenoid-quinone biosynthesis | Metabolism | 0.15384615 | 0.00328546 | 0.04448013 |
| Carbon fixation in photosynthetic organisms | Metabolism | 0.06818182 | 0.00314403 | 0.04448013 |
| HIF-1 signaling pathway | Environmental Information Processing | 0.03378378 | 0.00314073 | 0.04448013 |

### Table S10. Significantly enriched KEGG pathways in GSEA under the inflammatory condition

| **KEGG Term** | **NES** | **NOM p value** | **FDR Q value** |
| --- | --- | --- | --- |
| Taste transduction | 1.9251444 | 0 | 0.0300125 |
| Glycolysis / Gluconeogenesis | 1.9142126 | 0 | 0.0171903 |
| Starch and sucrose metabolism | 1.6275507 | 0.0182149 | 0.2218369 |
| Notch signaling pathway | 1.6018268 | 0.0159292 | 0.209035 |
| Cell adhesion molecules (CAMs) | 1.529191 | 0.0055046 | 0.3150523 |
| Systemic lupus erythematosus | 1.5175827 | 0.0088183 | 0.2899814 |
| Fructose and mannose metabolism | 1.5123092 | 0.0297398 | 0.2604321 |
| Glycosaminoglycan degradation | 1.4627165 | 0.06917 | 0.3378215 |
| PPAR signaling pathway | 1.4471135 | 0.0346715 | 0.3374104 |
| Type I diabetes mellitus | 1.4271291 | 0.0478821 | 0.3520054 |
| Pentose phosphate pathway | 1.3859884 | 0.077821 | 0.4290621 |
| Neuroactive ligand-receptor interaction | 1.3583128 | 0.0185497 | 0.4680809 |
| Leukocyte transendothelial migration | 1.3389673 | 0.0622896 | 0.4914933 |
| Leishmania infection | 1.3385148 | 0.066787 | 0.4574271 |
| Basal cell carcinoma | 1.3015219 | 0.0960452 | 0.5389935 |
| Olfactory transduction | 1.2830325 | 0.1059829 | 0.5647546 |
| Primary immunodeficiency | 1.2617955 | 0.1641509 | 0.6022743 |
| Cytokine-cytokine receptor interaction | 1.261175 | 0.0528109 | 0.5712479 |
| Inositol phosphate metabolism | 1.2261678 | 0.1657941 | 0.6599166 |
| Complement and coagulation cascades | 1.2195345 | 0.1752381 | 0.652048 |

### Table S11. Common DEGs under healthy condition and inflammatory condition

| **Gene ID** | **Gene Symbol** |
| --- | --- |
| 100652748 | 'TIMM23B' |
| 10397 | 'NDRG1' |
| 105373989 | 'LOC105373989' |
| 11069 | 'RAPGEF4' |
| 112268238 | 'LOC112268238' |
| 114112 | 'TXNRD3' |
| 115330 | 'GPR146' |
| 132158 | 'GLYCTK' |
| 151295 | 'SLC23A3' |
| 170384 | 'FUT11' |
| 1728 | 'NQO1' |
| 2026 | 'ENO2' |
| 2256 | 'FGF11' |
| 230 | 'ALDOC' |
| 26355 | 'FAM162A' |
| 2785 | 'GNG3' |
| 280664 | 'WFDC10B' |
| 2821 | 'GPI' |
| 285966 | 'TCAF2' |
| 29923 | 'HILPDA' |
| 29948 | 'OSGIN1' |
| 30001 | 'ERO1A' |
| 3006 | 'HIST1H1C' |
| 3012 | 'HIST1H2AE' |
| 3017 | 'HIST1H2BD' |
| 3077 | 'HFE' |
| 339105 | 'PRSS53' |
| 353322 | 'ANKRD37' |
| 3625 | 'INHBB' |
| 3952 | 'LEP' |
| 4282 | 'MIF' |
| 441054 | 'C4orf47' |
| 4601 | 'MXI1' |
| 5033 | 'P4HA1' |
| 51129 | 'ANGPTL4' |
| 5129 | 'CDK18' |
| 5143 | 'PDE4C' |
| 5190 | 'PEX6' |
| 5230 | 'PGK1' |
| 55076 | 'TMEM45A' |
| 55818 | 'KDM3A' |
| 55876 | 'GSDMB' |
| 55893 | 'ZNF395' |
| 5935 | 'RBM3' |
| 6515 | 'SLC2A3' |
| 664 | 'BNIP3' |
| 6676 | 'SPAG4' |
| 7167 | 'TPI1' |
| 7422 | 'VEGFA' |
| 79729 | 'SH3D21' |
| 8334 | 'HIST1H2AC' |
| 8351 | 'HIST1H3D' |
| 8357 | 'HIST1H3H' |
| 83882 | 'TSPAN10' |
| 8497 | 'PPFIA4' |
| 8553 | 'BHLHE40' |
| 8862 | 'APLN' |
| BGIG9606_37544 | 'BGIG9606_37544' |
| BGIG9606_54780 | 'BGIG9606_54780' |
